# Molecular and Functional Characterization of a *Trypanosoma cruzi* Nuclear Adenylate Kinase Isoform

**DOI:** 10.1371/journal.pntd.0002044

**Published:** 2013-02-07

**Authors:** María de los Milagros Cámara, León A. Bouvier, Gaspar E. Canepa, Mariana R. Miranda, Claudio A. Pereira

**Affiliations:** Laboratorio de Biología Molecular de Trypanosoma cruzi (LBMTC), Instituto de Investigaciones Médicas “Alfredo Lanari”, Universidad de Buenos Aires and CONICET, Buenos Aires, Argentina; Instituto de Investigaciones Biotecnológias, Argentina

## Abstract

*Trypanosoma cruzi*, the etiological agent of Chagas' disease, is an early divergent eukaryote in which control of gene expression relies mainly in post-transcriptional mechanisms. Transcription levels are globally up and down regulated during the transition between proliferating and non-proliferating life-cycle stages. In this work we characterized a nuclear adenylate kinase isoform (TcADKn) that is involved in ribosome biogenesis. Nuclear adenylate kinases have been recently described in a few organisms, being all related to RNA metabolism. Depending on active transcription and translation, TcADKn localizes in the nucleolus or the cytoplasm. A non-canonical nuclear localization signal was mapped towards the N-terminal of the protein, being the phosphate-binding loop essential for its localization. In addition, TcADKn nuclear exportation depends on the nuclear exportation adapter CRM1. TcADKn nuclear shuttling is governed by nutrient availability, oxidative stress and by the equivalent in *T. cruzi* of the mammalian TOR (Target of Rapamycin) pathway. One of the biological functions of TcADKn is ribosomal 18S RNA processing by direct interaction with ribosomal protein TcRps14. Finally, TcADKn expression is regulated by its 3′ UTR mRNA. Depending on extracellular conditions, cells modulate protein translation rates regulating ribosome biogenesis and nuclear adenylate kinases are probably key components in these processes.

## Author Summary

Infection with *Trypanosoma cruzi* produces a condition known as Chagas disease which affects at least 17 million people. Adenylate kinases, so called myokinases, are involved in a wide variety of processes, mainly related to their role in nucleotide interconversion and energy management. Recently, nuclear isoforms have been described in several organisms. This “atypical” isoform in terms of primary structure was associated to ribosomes biogenesis in yeast and to Cajal body organization in humans. Moreover nuclear adenylate kinases are essential for maintaining cellular homeostasis. In this manuscript we characterized *T. cruzi* nuclear adenylate kinase (TcADKn). TcADKn localizes in the nucleolus or cell cytoplasm. Nuclear shuttling mechanisms were also studied for the first time, being dependent on nutrient availability, oxidative stress and by the equivalent of the mammalian TOR pathway in *T. cruzi*. Furthermore we characterized the signals involved in nuclear importation and exportation processes. In addition, TcADKn expression levels are regulated at an mRNA level, being its 3′UTR involved in this process. These findings are the first steps in the understanding of ribosome processing in trypanosomatids.

## Introduction

*Trypanosoma cruzi*, the causative agent of Chagas' disease, is a protozoan parasite with a complex life cycle which involves two intermediary hosts, triatomine insects and mammals and three main parasite stages, epimastigotes and amastigotes which replicate in the insect vector and mammalian host respectively; and trypomastigotes the non-replicative form [1]. The complexity of its life cycle involves multiple morphological and metabolic changes that are possible due to a strict control of gene expression [2]. Early eukaryotes from the order Kinetoplastida, transcribe their genes as large polycistronic arrays and therefore rely on post-transcriptional mechanisms for gene expression regulation [3], [4], [5], [6], [7], [8], [9], [10].

Furthermore trypanosomatids present unique characteristics regarding ribosome structure [11], [12] and ribosomal locus organization [13]. Instead of having the typical ribosomal locus organization which consists of ribosomal promoter, ETS1 (external transcribed spacer 1), 18S rDNA, ITS1 (internal transcribed spacer 1), 5,8S rDNA, ITS2 (internal transcribed spacer 2), 28S rDNA, ETS2 (external transcribed spacer 2), ribosomal terminator and the 5S rDNA, they present the 28S rDNA fragmented in 7 small rDNAs [13]. There is almost no information about ribosome biogenesis in trypanosomatids, but their extremely divergent ribosomal locus suggests that they might present unique characteristics in ribosome biogenesis and assembly. For example in *T. brucei* ribosomal 5S rRNA biogenesis involves proteins which are exclusively found in trypanosomatids [14], [15], [16].

In the last few years, an atypical nuclear adenylate kinase (ADK, ATP∶AMP phosphotransferase, EC: 2.7.4.3) isoform has been characterized in several organisms, such as *Drosophila melanogaster* [17], *Saccharomyces cereviciae* (FAP7) [18], *Caenorhabditis elegans* [19] and *Homo sapiens* (hCINAP) [20], [21]. ADKs are mainly involved in maintaining the adenine nucleotide pool, which includes ATP synthesis from ADP [22]. They are distributed in all kind of organisms, from bacteria to vertebrates, presenting conserved motifs, structures and functions. However, nuclear ADKs present unique characteristics and differ enormously in terms of primary structure and function from other previously characterized ADKs. It has been shown that all nuclear ADKs, present phosphotransferase activity in vitro, furthermore the human and yeast variants also present ATPase activity [17], [19], [23], [24]. In *S. cereviciae*, FAP7 has shown several diverse functions; first of all it has been related to oxidative stress response by the activation of the transcription factor POS9 [18], secondly overexpression of FAP7 confers, resistance to arsenite exposure, a powerful oxidant [25], [26]. Finally FAP7 has been related to ribosome biogenesis; being involved in the final step of maturation of the 20S pre-rRNA, which corresponds to the cleavage at “site D” by direct interaction with Rps14, a ribosomal protein that is found near the 3′ end of the 18S rRNA [23], [25]. Interestingly, conserved residues predicted to be required for nucleoside triphosphate (NTP) hydrolysis are essential for FAP7 function in vivo [18], [23]. Furthermore the human isoform (hCINAP) has also been vastly characterized and is involved in Cajal body organization [27]; transcription process and cell cycle progression [28].

In trypanosomatids ADKs have been identified in *Leishmania* [29], *Trypanosoma* [30], [31] and *Phytomonas* [32]. In *T. brucei* [31] and *T. cruzi* [30] several isoforms have been characterized with different subcellular localization including, flagella, glycosomes, mitochondria, and cytoplasm [30], [31], [33], [34], mainly related to energy balance maintenance.

In the following work we characterized *T. cruzi* nuclear ADK isoform, showing that it is involved in ribosome processing and presents unique characteristics being completely different from the other isoforms found in these parasites.

## Materials and Methods

### Parasites

Stock cultures of *T. cruzi* epimastigotes of the Y strain were maintained in axenic conditions at 28°C in BHT (Brain Heart Triptose) media (pH 7) supplemented with 10% fetal calf serum, 100 U.mL^−1^ penicillin, and 100 mg.L^−1^ streptomycin [35]. Transfected parasites were maintained in the same media containing 500 µg.mL^−1^ of G418 and 10% fetal calf serum. Parasites were counted in a Neubauer hemocytometer chamber.

### Plasmid constructions and stable transgenic parasites lines

*T. cruzi* TcADKn gene (Systematic ID: Tc00.1047053507023.280) was amplified from genomic DNA of epimastigotes from the Y strain and cloned in the pRSET-A vector (Invitrogen) by digesting with *HindIII/XhoI* and TcRps14 (Systematic ID: Tc00.1047053506945.230) was amplified from genomic DNA of epimastigotes from the Y strain and cloned in the pGEX vector (GE Healthcare) digested with *BamHI/XhoI*. The sequence coding for full-length *T. cruzi* TcADKn was cloned in the pTEX-eGFP expression vector by digesting with *HindIII/SalI*. The pTEX-eGFP plasmid was constructed by cloning the eGFP into the pTEX-TAP vector, kindly provided by Dr. Esteban Serra (IBR, Rosario). A total of 10^8^ parasites of the Y strain, were grown in BHT medium at 28°C, harvested by centrifugation, washed with PBS, and resuspended in 0.35 mL of electroporation buffer (PBS containing 0.5 mM MgCl_2_, 0.1 mM CaCl_2_). The cell suspension was mixed with 50 µg of plasmid DNA in 0.2 cm gap cuvettes (Bio-Rad). Parasites were electroporated with a single pulse of 400 V, 500 µF with a time constant of about 5 ms. Stable cell lines were achieved after 30 days of treatment with 500 µg.mL^−1^ G418 (Sigma) [36]. For deletion analyses TcADKn segments were amplified by PCR from the pTEX-ADKn-eGFP plasmid, cloned into pGEM T-easy vector (Promega) and subcloned in the pTEX-OMNI-eGFP vector. The pTEX-OMNI vector derives from the pTEX-GFP vector [37], by the addition of the 3-FLAG (Sigma), HA (influenza virus hemagglutinin), and aT (C-terminal alpha tubulin) epitopes present in the pDIY cloning vector (GI:374430409). TcADKn locus was amplified from genomic DNA, the 3′ UTR was cloned in the pTREX-OMNI-eGFP vector which contains the same epitopes as the pTEX-OMNI vector but in a pTREX backbone. The pTEX-Dhh1-eGFP plasmid was kindly provided by Dr. Alejandro Cassola (IIB-UNSAM). For oligonucleotide sequence refer to Table S2.

### Fluorescence microscopy

Freshly grown trypanosome samples were washed twice in PBS. After letting the cells settle for 30 min at room temperature in poly-L-lysine coated coverslips, parasites were fixed at room temperature for 20 min with 2% formaldehyde in PBS, followed by a cold methanol treatment for 5 min. Afterwards, all the samples were treated with anti-TcADKn (1∶200), anti-GFP antibody (1∶500) (Invitrogen) or anti-TcPABP1 (1∶500) for 1 h followed by 1 h incubation with anti-mouse (daylight 488, Jackson Immuno Research) or anti-rabbit (daylight 594, Jackson Immuno Research) secondary antibody. Slides were mounted using Vectashield with DAPI (Vector Laboratories). Cells were observed in an Olympus BX51 fluorescence microscope. Images were recorded with an Olympus XM10 camera. Images were analyzed with MBF ImageJ for microscopy bundle.

### Protein expression and purification

Fusion proteins were expressed in *Escherichia coli* BL21 (DE3) or DH5α. Cells were grown in Luria broth medium (LB) at 37°C with ampicillin to an optical density of 0.4 to 0.5 measured at 600 nm (OD_600_). Protein expression was induced with 1 mM of isopropyl-β-D-thiogalactoside (IPTG) for 16 to 20 h at 37°C. Cells were harvested by centrifugation, and pellets were resuspended in 5 to 8 volumes of breaking buffer (350 mM NaCl, 50 mM Tris-HCl, pH 7.4, 0.5 mM EDTA) containing a protease inhibitor PMSF (10 µg/ml) and disrupted by sonication. Extracts were clarified by centrifugation at 12,000 rpm for 20 minutes. To purify recombinant 6x-His-TcADKn protein, clarified extracts were incubated with Ni-nitrilotriacetic acid beads (1 mL beads for 5 g of cell pellet; QIAGEN) for 12 h at 4°C. Proteins were eluted with 5 bead volumes of breaking buffer containing 200 mM imidazole.

Eluates containing nearly homogenous recombinant protein were pooled and dialyzed overnight in breaking buffer containing 20% glycerol and stored at −80°C. This procedure yielded 90%-pure recombinant protein, as judged by SDS-polyacrylamide gel electrophoresis (SDS-PAGE) and Coomassie brilliant blue staining. To purify glutathione S-transferase (GST) fusion proteins, GST-TcRps14 and the GST epitope, clarified extracts were incubated with 2 ml of glutathione-Sepharose beads (Amersham) for 4 h at 4°C. The beads were washed extensively with breaking buffer, and proteins were eluted with the same buffer containing 20 mM of reduced glutathione (Amersham). Eluates were dialyzed overnight in breaking buffer containing 20% glycerol and stored at −80°C.

### Enzyme assays

For ADK activity, 50 µg of purified recombinant protein fraction were added to the reaction mixture (100 mM Tris-HCl buffer pH 7.5, 20 mM glucose, 5 mM MgCl_2_, 100 mM KCl, 2 mM dithiothreitol, 1 mM NADP^+^, 5 U.mL^−1^ and 2 U.mL^−1^ glucose-6-phosphate dehydrogenase) in a cuvette in a final volume of 0.5 mL. After 5 min incubation at 35°C the reaction was started by the addition of a small volume of ADP to a final concentration of 10 mM. ADK activity was calculated by measuring the increase in absorbance at 340 nm that accompanied the reduction of NADP+ [30]. For ATPase activity a sample of 50 µg of protein was added to the reaction mixture (100 mM Tris-HCl, pH 7.5, 60 mM KCl, 5 mM MgCl_2_, 5 U.mL^−1^ of polynucleotide kinase, 5 U.mL^−1^ of lactate dehydrogenase, 20 mM phosphoenolpyruvate, 1 mM NADH). After 5 min incubation at 35°C the reaction was started by the addition of a small volume of ATP. ATPase activity was calculated by measuring the decrease in absorbance at 430 nm that accompanied the oxidation of NADH. The results were plotted and the slope was used to calculate specific activities.

### RNA extraction and real time PCR assays

1×10^8^ epimastigotes, were harvested by centrifugation, washed with PBS, resuspended in 1 mL of Tri-Reagent (Sigma), mixed by inversion and 200 µL of chloroform were added followed by centrifugation at 12,000×g at 4°C. The supernatant was transferred to a clean test tube with 500 µL of isopropanol, after 10 min incubation at room temperature; they were centrifuged at 12,000×g for 15 min at 4°C. The pellet was washed with ethanol 75%, left to dry and resuspended in 20 µL of RNase-free water. RNA concentrations were determined spectrophotometrically, purity was confirmed by gel electrophoresis. 3 µg of RNA were used for retrotranscription, which were previously treated with DNaseI (Sigma) in order to eliminate any DNA contamination. TcADKn mRNAs were isolated by RT-PCR cloned in pGEM T-easy vector (Promega) and sequenced. TcADKn differential mRNA expression along the epimastigote's growth curve was quantified by SYBR green-based real-time PCR in a Real-Time PCR system (Bio-Rad) using default protocols. Data were relativized to 18S expression. Three independent experiments were carried out. eGFP expression along the growth curve of parasites expressing the pTREX-OMNI-eGFP-LAN or pTREX-OMNI-eGFP constructions was quantified by SYBR green-based real-time PCR in a Real-Time PCR system (Bio-Rad) using default protocols. GFP expression was relativized to neomycin (Neo) expression (present in the pTREX-OMNI-eGFP vector). Three independent experiments were carried out.

### Immunoprecipitations assays

1.25×10^8^ epimastigotes from day 2 of culture were harvested washed with PBS, resuspended in buffer A (20 mM Tris-HCl, pH 7.6, 2 mM MgCl_2_, glycerol 10%, Nonidet P-40 0.5%, 1 mM EDTA, 1 mM DTT, 0.25 M sacarose, 50 mM KCl, 1 mM E64 and RNAse inhibitor from Sigma) and incubated for 30 min in ice. They were harvested at 10,000×g for 15 min at 4°C, the supernatant was transferred to a clean tube containing 20 mg of protein G-agarose (Sigma) and 10 µl of preimmune serum the mixture was left in agitation for 1 h at 4°C. This fraction corresponded to the clarified extract. In parallel 20 mg of protein G-agarose were blocked with 100 µg of BSA in buffer A for 2 h at room temperature. After the pre-blocking 10 µl of anti-TcADKn serum were added and incubated for 2 h at 4°C, washed three times with 500 µL of buffer A and 400 µL of clarified epimastigotes extract was added and left in agitation for 1 h at 4°C. After incubation beads were washed three times with buffer A and five times with PBS 1×. The pellet was resuspended in 800 µL of TriReagent (Sigma) for RNA extraction. A small fraction was separated for protein analysis.

### Protein-protein interaction

10 µg of recombinant 6x-His-tagged *T. cruzi* arginine kinase (TcAK, Tc00.1047053507241.30) and 6x-His-tagged TcADKn were incubated with 10 µg of GST or 5 µg of GST-TcRps14, in buffer K (150 mM NaCl, 50 mM Tris-HCl pH 7.4, 0.5 mM EDTA) containing protease inhibitor PMSF (10 µg.mL^−1^) in a final volume of 30 µL. After 1 h of incubation in ice, 190 µL of buffer K were added to the mixtures, 20 µL were removed for analysis (10% of the input), and the remainder was incubated with 10 µL of glutathione-Sepharose beads (Amersham) for 1 h in ice with regular agitation. Beads were spinned down by centrifugation and 20 µL of the supernatant were subsequently removed for analysis. The beads were washed three times with 1 mL of ice-cold buffer K. Bound proteins were extracted by boiling the beads in SDS-PAGE loading buffer (output) and resolved in 12% polyacrylamide denaturing gels. Proteins were identified by Western Blot analysis.

### Protein detection

Western Blots were performed using total *T. cruzi* extracts fractioned by electrophoresis in polyacrylamide denaturing gels and transferred to polyvinylidene fluoride (PVDF) membranes. The PVDF membranes were treated for 1 h with 5% non-fat dry milk in PBS and then incubated with the primary antibody ON, using anti-TcADKn diluted 1∶5000, anti-His 1∶3000 (Sigma) or anti-GST diluted 1∶2000 (Invitrogen), anti-GFP diluted 1∶2500 and anti-α-tubulin diluted 1∶2000 (Abcam). Membranes were washed and incubated with the corresponding secondary antibody for two hours (anti-mouse HRP 1∶2500, anti-rabbit HRP 1∶2500, Vector Labs). Detection was done by chemiluminescence (Pierce).

### Yeast transformation

The 3HA-FAP 7 strain (mat α ura3-52 lys2-80 ade2-101 trp163 his3-200 leu2-1) was kindly provided by Dr. Baserga. Strains were grown in YPG (1% yeast extract, 2% peptone, 2% galactose) until transformation. Yeasts were transformed as explained in http://home.cc.umanitoba.ca/~gietz/. The genes of TcADKn, TbADKn, FAP7, *E. coli* ADK, TcADK6, TbADKF were amplified and cloned in the p416 vector [38], kindly provided by Dr. Cecilia D'Alessio, Fundacion Instituto Leloir. Transformed yeast were grown in minimum medium with galactose for selection and afterwards shifted to minimum medium with glucose for complementation assays [23].

### Drug and differential media treatments

Exponentially growing *T. cruzi* epimastigotes were treated with different drugs: actinomycin D (Sigma) 10 µg.mL^−1^ for 4 h, cicloheximide (Sigma) 50 µg.mL^−1^ for 4 h, puromycin 200 µg.mL^−1^ 4 h, starvation in PBS 24 h, leptomycin B (Sigma) 0.1 µg.mL^−1^ for 5 h, rapamycin (Sigma) 100 µM for 6–8 h, phleomycin 150 µg.mL^−1^ for 4 h, hydrogen peroxide 200 µM for 1 h. After leptomycin and rapamycin treatment fluorescence was quantified for forty treated and untreated parasites. In each parasite the fluorescence from the green (GFP) channel was quantified in an area selected according to blue signal (DAPI) fluorescence (nucleus) using the RGB plugin in ImageJ (http://rsb.info.nih.gov/ij/). Cytoplasmic fluorescence was quantified in the same way selecting the brightest perinuclear areas in the green (GFP) channel. Selection criterion was the same for all transfected parasites. Cytoplasmic fluorescence determinations included the previously selected nuclear areas and fluorescence values, which were afterward subtracted, resulting in the area and values corresponding to the cytoplasm. For each parasite, the relationship between fluorescence/area was obtained for the nucleus and cytoplasm and then the ratio between the nuclear and cytoplasmic values was calculated. For media supplementation experiments BHT medium of parasites in day 10 of culture was supplemented with glucose or proline 2% and were incubated for 24 h. Results were monitored by immunofluorescence. For RNAseI treatment, epimastigotes in day 2 of culture, were harvested, washed with PBS, resuspended in buffer (50 mM Tris-HCl pH 7.8) and broken with liquid nitrogen, samples were treated for 2 h at 37°C with 20 mg.mL^−1^ of RNaseI. Samples were boiled in SDS-PAGE loading dye and analyzed by Western Blot. For native gel analysis, loading buffer (Tris-HCl 100 mM pH 8, sucrose 2%, BPB 0,05%) was added to protein samples and analyzed by Western Blot.

### Cloning and identification of 18S precursors

18S rRNA precursors were isolated using a simple adapter ligation protocol. A standard oligonucleotide blocked in its 5′ end was adenylated using the New England Biolabs (NEB) adenylation kit following manufacturer's indications. Epimastigotes RNA was extracted as explained above, for each ligation reaction three RNA samples were pooled. The 3′ adenylated adapter was ligated in the absence of ATP using the T4 RNA ligase 2 truncated (NEB) using manufacturer's indications. The final ligation products were reversely transcribed into cDNA using a complementary oligonucleotide to the adapter. PCRs were performed using specific primers for the 18S and ITS region. PCR products were cloned in the pGEM T-easy vector (Promega) and submitted for sequencing. The same strategy was used for RNA extracted from immunoprecipitates against TcADKn. RT PCR were done using oligonucleotides, for the ITS region, TcH2B (Systematic ID: Tc00.1047053511635.20) and TcNDPK3 (Systematic ID: Tc00.1047053510879.210). A standard PCR protocol was used, 5 minute denaturation at 95°C and 30 cycles: 1 minute denaturation at 95°C, 1 minute at the corresponding annealing temperature, and 1 minute at 72°C; finally 10 minutes at 72°C. Controls without retrotranscriptase were done to eliminate any DNA contamination possibilities.

### Gene identification

Sequences were obtained from the TriTrypDB (http://tritrypdb.org/tritrypdb/). Assembly and sequence data analysis, including ORFs prediction, were carried out using the software package Vector NTI 10.3.0 (Invitrogen) and the online version of BLAST at the NCBI (http://blast.ncbi.nlm.nih.gov/Blast.cgi). Sequence analysis and nuclear localization signals and nuclear exportation signal were carried out using the online predictors http://www.psort.org/ [39], http://www.cbs.dtu.dk/services/NetNES/,and http://psort.hgc.jp/, respectively [40].

## Results

### Characterization of *T. cruzi* nuclear adenylate kinase

#### Identification of *T. cruzi* nuclear ADK (TcADKn)

Nuclear ADKs have been studied in the last few years in humans, yeasts, fruit flies and worms [17], [18], [19], [20], [21]. By genome data mining in the TriTryp database (http://tritrypdb.org/), using the sequences of the previously characterized nuclear ADKs as baits, we found putative nuclear ADK genes in the genomes of *T. cruzi*, *T. brucei* and *L. major* (Systematic IDs: Tc00.1047053507023.280, Tb927.6.3210 and LmjF30.1890, respectively). As expected, they all presented the P-loop domain and showed approximately 45% of amino acid similarity between each other and with the other nuclear ADKs previously reported (Figure S1A, Table S1). Sequence analysis revealed that they did not present canonical nuclear localization signals (NLS); TcADKn presented a leucine-rich region towards the C-terminal of the protein, similar to the typical nuclear exportation signals (NES) that are recognized by the CRM1 transporter (Chromosome Region Maintenance 1 protein, or Exportin-1) [41]. Furthermore TcADKn amino acid sequence is highly divergent from the other six isoforms of ADKs previously characterized in *T. cruzi*, which is clearly reflected in the low similarity and identity percentages (less than 30%) between each other (Figure S1B, Table S1)[30].

#### TcADKn has ADK and ATPase activities

TcADKn gene was amplified by PCR, cloned and expressed in *E. coli*. TcADKn gene was amplified by PCR, cloned and expressed in *E. coli* for biochemical enzyme assays and for antibodies' production (Figure S2). Recombinant TcADKn is a *bona fide* ADK because it presented biochemical activity using ADP as substrate (specific activity 0,2 nmol.min^−1^.mg^−1^) (Figure S3A); in addition, it was capable of hydrolyzing ATP in the absence of AMP, characteristic of ATPase activity (specific activity 0,34 nmol.min^−1^.mg^−1^) (Figure S3B). A TcADKn P-loop mutant was also generated; when the conserved lysine was replaced for an arginine, an amino acid of similar characteristics, TcADKn lost both ADK and ATPase activities highlighting the importance of this residue for the catalysis [42], [43].

#### TcADKn expression and localization along *T. cruzi* life cycle

**Figure 1:**
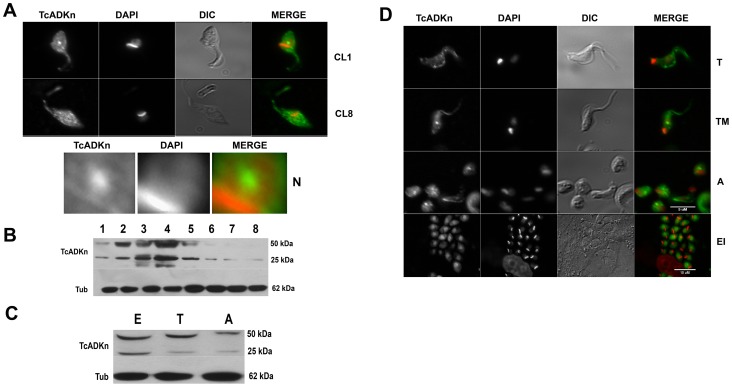
Tc ADKn expression in the different stages of *T. cruzi*. A) TcADKn localization in epimastigotes of *T. cruzi* along the growth curve. Parasites were grown BHT medium, starting from 1×10^6^ epimastigotes, samples were collected at day 1 of culture (CL1) and day 8 (stationary phase, CL8). TcADKn localization was followed by indirect immunofluorescence using specific anti-TcADKn mouse generated antibodies. Nuclear are was enlarged for nucleolar visualization (N). B) Western Blot analysis using anti-TcADKn antibodies, to study its expression along the epimastigotes growth curve. *T. cruzi* epimastigotes were grown in BHT medium, starting from 1×10^6^ total parasites; samples were collected daily for protein sample preparation. In each lane 4×10^6^ parasites were loaded. C) Western Blot analysis in the three main stages of *T. cruzi* life cycle epimastigotes, trypomastigotes and amastigotes. In every Western Blot, tubulin (TUB) was used as loading control. In each lane 4×10^6^ parasites were loaded. D) Indirect immunofluorescence to study TcADKn localization in mature trypomastigotes (T), metacyclic trypomastigotes (TM), amastigotes (A), intracellular epimastigotes (EI). Infective stages were obtained from infection of VERO cells, trypomastigotes (T) were collected daily 5 days post-infection, and amastigotes (A) were collected 10 days post-infection. Intracellular epimastigotes (EI) were obtained 7 days post-infection.

*Trypanosoma cruzi* epimastigotes have to adapt their metabolism from a high-nutrient medium to a low one as they travel through the alimentary canal of the insect host. During this process gene expression is globally modified [44] a phenomenon which can be mimicked in vitro [45]. In order to study TcADKn subcellular localization and expression we performed indirect immunofluorescence microscopy and western blot assays along the epimastigote's growth curve and infective stages of the parasite. In 90% of the analyzed parasites TcADKn presented a typical nucleolar and granular cytoplasmic distribution in epimastigotes during the first days of culture. Nucleolar localization was lost as parasites reached the stationary growth phase; after day 7 of culture nucleolar localization was detected in less than 10% of the parasites (Figure 1A). Nucleolar localization of TcADKn is evident upon enlargement of the nuclear area (Figure 1AN). Furthermore, we also detected strong TcADKn signal in epimastigotes flagella. Protein migration from the nucleolus to the flagella has been previously described in other lower eukaryotes [46].

Furthermore protein expression analysis revealed that total TcADKn levels decreased along the epimastigote's growth curve, after reaching its highest expression peak in day 4 of culture. A higher molecular weight protein band (50 KDa) was also detected which could indicate that TcADKn suffers a posttranslational modification (Figure 1B).

In addition, we studied TcADKn's expression in the infective stages of the parasite, being practically the same in the main stages of the parasite (Figure 1C). Nucleolar signal was detected in amastigotes, metacyclic trypomastigotes and intracellular epimastigotes (Figure 1D). In mature bloodstream trypomastigotes, where there is no nucleolus [47], TcADKn was completely cytoplasmic concentrating in parasites flagella (Figure 1D).

### TcADKn's nuclear shuttling

#### TcADKn presents a non-canonical nuclear localization signal

**Figure 2:**
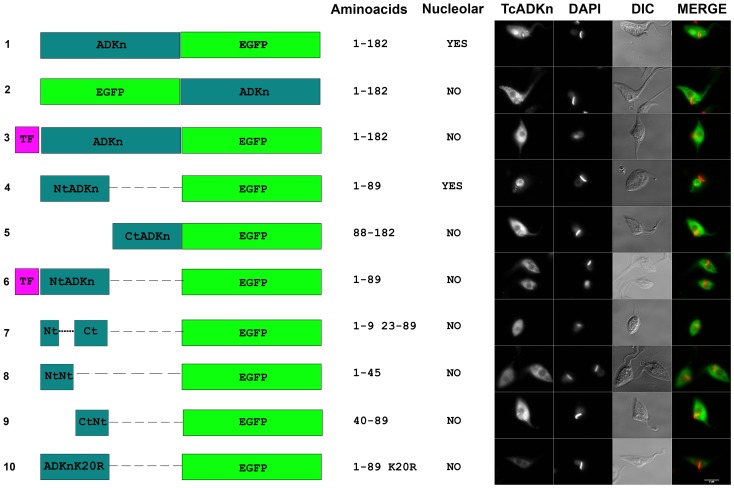
TcADKn nuclear localization signal. Deletion analysis and GFP fusions were built in order to determine the sequence responsible for TcADKn nuclear localization. The scheme on the left side shows the GFP fusions, indicating TcADKn amino acids range fused to GFP (Amino acids) and the resulting subcellular localization (Nuclear). *T. cruzi* epimastigotes were transfected with the constructions and fluorescence was followed by fluorescence microscopy NtADKn represents aminoterminal of the protein (1–89 amino acids), CtADKn represents the carboxiterminal of the protein (88–182 amino acids), NtNtADKn represents the aminoterminal of Nt (1–45 amino acids), Nt–Ct represents NtADKn were the p-loop has been deleted (1–9, 23–89 amino acids), CtNtADKn represents the carboxiterminal of NtADKn (40–89 amino acids). The non-classical NLS was mapped to the N-terminal (Nt) of the protein, being the catalytic lysine of the p-loop (K20) essential for its localization (ADKnK20R). Images corresponding to TcADKn, 4′,6-diamidino-2-phenylindole (DAPI), differential interference contrast microscopy (DIC) and the merged images, are showed in the right panel.

As it was explained above by bioinformatics analysis we were not able to detect the presence of classical nuclear localization signals in TcADKn sequence. In order to determine the non-classical nuclear localization signal we followed a deletion analysis strategy followed by heterologous eGFP fusion protein expression in *T. cruzi* epimastigotes (Figure 2). Over expression levels were high, consequently in most of the cases nucleolar localization was lost being dispersed all over the nucleus, similar results have been described for other nucleolar proteins in trypanosomatids [48]. We observed nuclear localization in transgenic parasites carrying the eGFP fused to the C-terminal of full-length TcADKn (TcADKn-eGFP). Similar results were observed in parasites expressing the eGFP fused to the C-terminal of the N-terminal portion of the protein (NtTcADKn-eGFP). Nuclear localization was lost, being completely cytoplasmic in parasites expressing the fusion protein which carried the eGFP fused to the N-terminal of full-length TcADKn (eGFP-TcADKn). Cytoplasmic localization was also observed when the tri-FLAG epitope was added towards the N-terminal of the constructions TcADKn-eGFP and Nt-TcADKn- eGFP (TriFLAG-ADKn-eGFP and triFLAG-NtADKn-eGFP). Furthermore, cytoplasmic signal was observed when the P-loop was deleted from the NtADKn-eGFP construction, indicating that this conserved domain is necessary for nuclear localization. Similar results were obtained when a single point mutation was done, in which the conserved lysine of the P-loop was modified for an arginine (Nt(K20R)ADKn-eGFP). The presented data suggests that the non-canonical nuclear localization signal would be present towards the N-terminal of the protein, being the P-loop essential for its localization. This region could be involved in protein-protein interaction with the nuclear importation complex, highlighting the importance of this domain, not only for being essential for enzymatic activity but also for its critical role in TcADKn localization.

#### TcADKn nuclear exportation could be dependent of the CRM1 pathway

**Figure 3:**
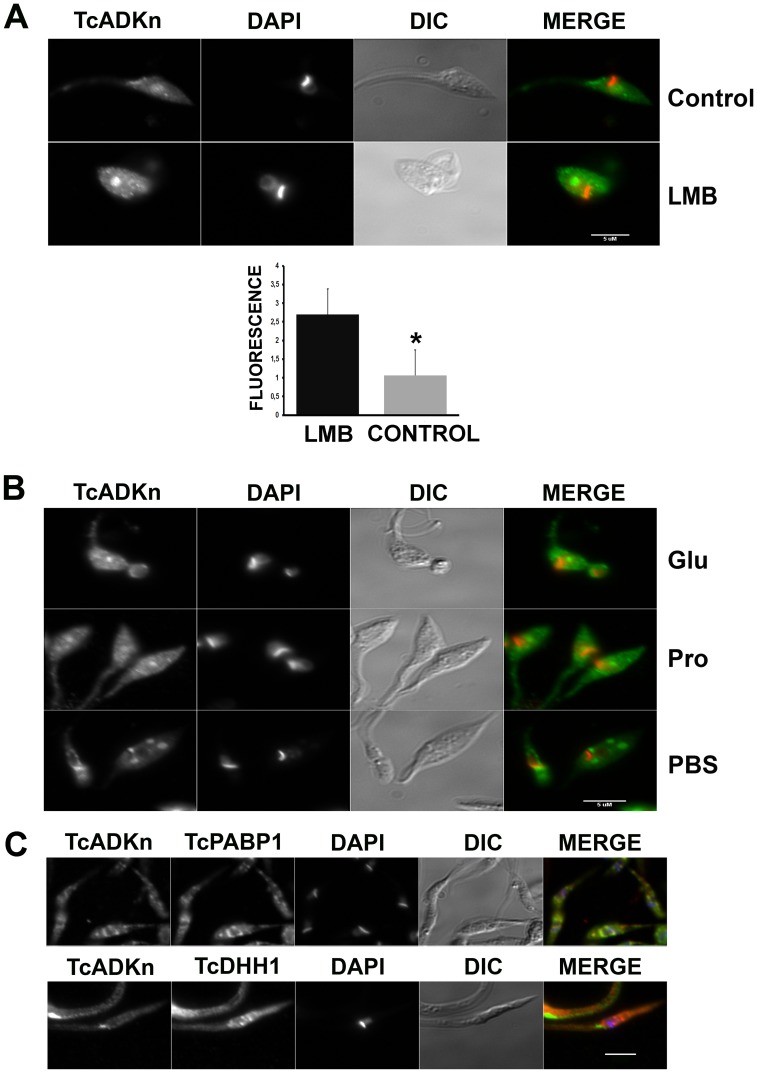
TcADKn nuclear exportation and nutrient availability regulation. A) *T. cruzi* Epimastigotes in exponential growth phase were treated with leptomycin B, 0.1 µg.mL^−1^ for 5 h (LMB), results were analyzed by immunofluorescence and fluorescence was quantified for 40 treated and untreated parasites. In each parasite the fluorescence from the green channel (GFP) was quantified in an area selected according to blue signal (DAPI) fluorescence (nucleus) using the RGB plugin in ImageJ (http://rsb.info.nih.gov/ij). Cytoplasmic fluorescence was quantified in the same way selecting the brightest perinuclear areas in the green channel (GFP). Selection criterion was the same for all transfected parasites. Bars represent mean ± S.D. Statistically significant difference was calculated by t-student test (p<0,05). B) *T. cruzi* epimastigotes in stationary growing phase in BHT medium, were supplemented with glucose (Glu) or proline (Pro) 2% for 12 h. Epimastigotes from day 2 of culture were maintained in starvation conditions (PBS) for 24 h. Data were analyzed by indirect immunofluorescence using anti-TcADKn antibodies. C) Epimastigotes from day 2 of culture were maintained in starvation conditions (PBS) for 24 followed by indirect immunofluorescence for colocalization with p-bodies (TcDhh1) and stress granules markers (TcPABP1).

Bioinformatic analysis revealed that TcADKn has a putative nuclear exportation signal (63% of probability using the NETNES1.1 predictor) which is recognized by the nuclear exportation adapter CRM1 (Chromosome Region Maintenance 1 protein, or Exportin-1). This transporter has been characterized in several organisms including *T. cruzi* and is inhibited by leptomycin B (LMB) [49]. Epimastigotes in exponential growth phase were incubated with LMB for 5 hours, and the results were analyzed by immunofluorescence assays, followed by fluorescence quantification. Nuclear fluorescence was quantified and normalized, revealing that treated parasites presented about 2.5-times fold more fluorescence than untreated ones (p<0,05) (Figure 3 A). Hence, TcADKn nuclear exportation could be dependent of the CRM1 transporter being its transport energy dependent [49].

### TcADKn nuclear localization regulation

#### TcADKn nucleolar localization is dependent on nutrient availability

One of the most important changes occurring along the parasites growth curve is nutrient availability, which decreases accompanying culture days. In order to study if TcADKn nuclear localization was dependent on this variable, we supplied media of epimastigotes in stationary phase with either one of the major carbon sources used by parasites along their life cycle, glucose (2%) or proline (2%) [50], [51], [52]. Interestingly, after a 24 h treatment we observed that TcADKn shuttled from the cytoplasm to the nucleous. Complementary to the previous experiment, we tested if upon nutrient deficiency TcADKn could shuttle from the nucleus to the cytoplasm of exponentially growing parasites. After shifting freshly grown epimastigotes from BHT media to PBS for 24 h we observed that nucleolar localization was lost and that TcADKn localized in cytoplasmic granules (Figure 3B). These granules co-localized with the stress granules marker (PABP1, polyadenylate-binding protein 1) but not with the P-bodies marker (Dhh1, ATP dependent RNA helicase), both type of granules correspond to protein and mRNA silencing structures that arise under stressful conditions such as low nutrient availability and oxidative damage (Figure 3C) [53], [54].

#### TcADKn nucleolar localization is dependent on active transcription and ribosome assembly

**Figure 4:**
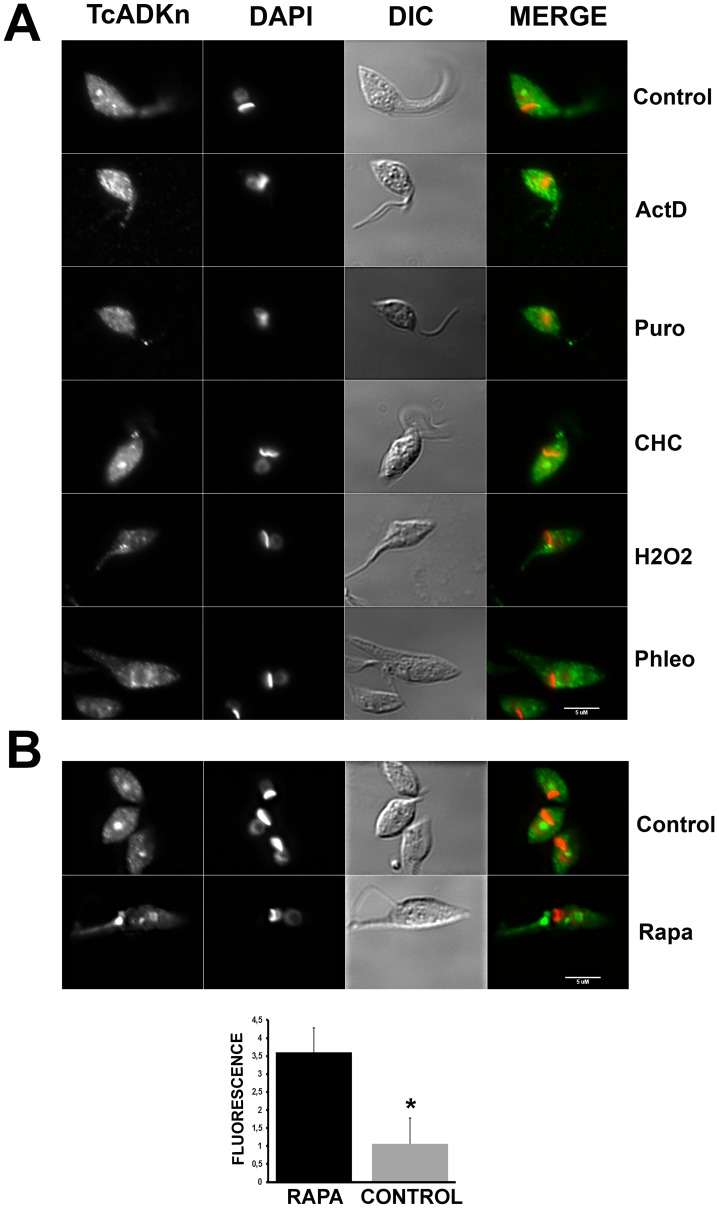
Regulation of TcADKn nucleolar localization. A) Fluorescence microscopy of *T. cruzi* epimastigotes in exponential growth phase, treated with Control (control), actinomycin D 10 µg.mL^−1^ for 4 h, (ActD), puromycin 200 µg.mL^−1^ for 4 h (Puro), cycloheximide 50 µg.mL^−1^ for 4 h (CHC), rapamycin 100 µM 12 h (Rapa), hydrogen peroxide 200 µM for 1 h (H_2_O_2_) and phleomycin 100 µg.mL^−1^ 1 hour (Phleo). Drugs were provided by Sigma. B) For rapamycin treatment, fluorescence was quantified for 40 treated and untreated parasites. In each parasite the fluorescence from the green channel (GFP) was quantified in an area selected according to blue signal (DAPI) fluorescence (nucleus) using the RGB plugin in ImageJ (http://rsb.info.nih.gov/ij). Cytoplasmic fluorescence was quantified in the same way selecting the brightest perinuclear areas in the green channel (GFP). Selection criterion was the same for all transfected parasites. Bars represent mean ± S.D. Statistically significant difference was calculated by t-student test (p<0,05).

In trypanosomatids it has been determined that ribosomal biogenesis and protein translation diminish as parasites reach the stationary phase [55]. Considering these data, the next issue we intended to answer was if TcADKn subcellular localization was under the control of active transcription and translation processes. To elucidate this question we performed treatments with transcription inhibitor actinomycin D (ActD), and two protein translation inhibitors, puromycin (PURO), which causes premature chain release and cycloheximide (CHC) which exerts its effect by interfering with the translocation step in protein synthesis, blocking translational elongation [56], [57], [58], [59]. After drug incubations we observed that nucleolar localization was lost upon cycloheximide and actinomycin D treatment, then again TcADKn concentrated in cytoplasmic granules. On the contrary, puromycin treatment did not affect nucleolar localization (Figure 4A). These results suggest that TcADKn localization could be under the regulation of transcription and translation processes.

#### TcADKn nucleolar localization is lost upon oxidative stress

Several nucleolar proteins change their localization upon cellular homeostasis disruption [60], [61]. A clear example of this is the oxidative stress response in which protein reorganization has also been described in trypanosomatids [62].

In order to study if TcADKn was involved in the oxidative stress response we challenged *T. cruzi* epimastigotes with sub-lethal concentrations of hydrogen peroxide (200 µM) [63]. Nucleolar localization was completely lost and cytoplasmic granules were observed after one hour treatment (Figure 4A, H_2_0_2_). These results indicate that TcADKn nucleolar localization could also be under the regulation of the oxidative damage response.

#### TcADKn concentrates in perinuclear areas after DNA damage

The localization of a vast number of nucleolar proteins is governed by DNA integrity [64], [65]. Being TcADKn a nucleolar protein we studied its localization after generating DNA damage by treating exponentially growing *T. cruzi* epimastigotes with phleomycin. After a three hour treatment we observed that TcADKn nucleolar localization was lost and it concentrated in perinuclear areas (Figure 4A, Phleo). This could be due to the arrest in transcription levels that occur as a consequence of DNA damage [66].

#### TcADKn nucleolar localization could be dependent on the TOR pathway

In the last few years the TOR (Target of Rapamycin) signaling pathway has been characterized in several organisms including trypanosomatids [67]. This pathway has been related to nutrient availability sensing and regulates the expression and localization of several ribosomal proteins [68], [69]. Since TcADKn nuclear localization and expression levels are controlled by nutrient availability, the next issue we approached was if TcADKn was controlled by the TOR pathway, *T. cruzi* epimastigotes in exponential growth phase were incubated with rapamycin an inhibitor of TORC-1 complex [70]. After treatment we observed that TcADKn concentrated in the nucleus of treated parasites (Figure 4B, Rapa). Nuclear fluorescence was quantified and data were normalized to DAPI fluorescence. Treated parasites presented 3.3-times fold more fluorescence in the nucleus than untreated parasites (p<0,05). The results suggest that TcADKn could be under the control of the TOR pathway, principally related to nutrient availability. However, little is known about this pathway in trypanosomatids so further experiments are required to confirm these results.

### Physiological functions of TcADKn

#### TcADKn is able to complement FAP7 in yeast

**Figure 5:**
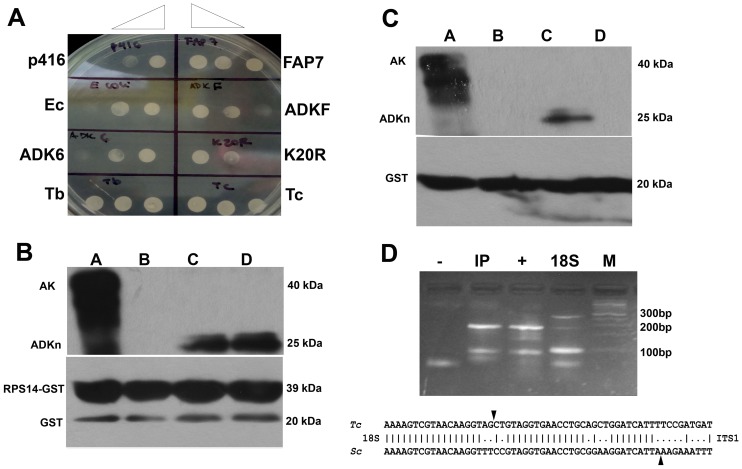
TcADKn role in ribosome biogenesis. A) Yeast complementation assay, Gal HA-FAP7 yeast strain were transformed with TcADKn, TcADKn(K20R), TbADKn, TbADKF (*T. brucei* mitochondrial isoform), *E. coli* ADK, TcADK6 (*T. cruzi* mitochondrial isoform) and ScFAP7. After transformation yeast were shifted to selective minimum medium containing glucose. Only yeast carrying TbADKn and TcADKn were able to grow in selective medium. B) In vitro protein interaction assays between recombinant 6xHisTcADKn, 6xHisTcAK and GST-TcRps14. Lane A and B input and output of the interaction assay between 6-His-TcAK and GST-TcRps14, lanes C and D interaction between TcRps14GST- HisTcADKn. Results were analyzed by Western Blot using monoclonal anti-HIS and anti-GST antibodies. C) In vitro protein interaction assays, inputs and outputs respectively between, lanes A and B recombinant 6xHisTcAK and the GST tag, lanes C and D 6xHisTcADKn and the GST tag. Results were analyzed by Western Blot using monoclonal anti-HIS and anti-GST antibodies. D) 18S precursors were isolated using a microRNA isolating system. Total RNA were ligated to an adenylated DNA adapter, followed by cDNA generation and RT-PCR using specific primers for the 18S and for the DNA adapter. Data were analyzed by agarose electrophoresis. Sequence alignment of 18S precursors of site D found in TcADKn immunoprecipitates and the precursor of *S. cereviciae*.

As it was mentioned before, FAP7, the yeast nuclear ADK isoform, has been related to ribosome processing [29]. Since FAP7 is essential for cell growth, a conditional allele in which a chromosomal N-terminal triple-HA-tagged FAP7 allele was placed under the control of a galactose inducible/dextrose-repressible promoter (GAL::3HA-FAP7) was generated [23]. In order to study if TcADKn could be involved in similar processes we performed yeast complementation assays in the Gal-3HA-FAP7 strain; we also included the following controls: p416 empty vector, FAP7, TbADKn (*T. brucei* ortholog of TcADKn), TcADK6 (putative *T. cruzi* mitochondrial ADK isoform), *E. coli* ADK, TbADKF (putative *T. brucei* mitochondrial ADK isoform) and TcADKn nonfunctional mutant (K20R). After transformation and selection in galactose minimum medium (Figure S4) yeast were shifted to selective minimum medium with glucose. We observed that only TcADKn and TbADKn were able to rescue the lethal phenotype observed in FAP7 depleted yeast (Figure 5A). These results indicate that TcADKn (and TbADKn) could be involved in ribosome biogenesis and that the conserved lysine of the P-loop would be critical for its function.

#### TcADKn interacts with TcRps14 in vitro

In yeast FAP7 interacts with Rps14 (40S ribosomal protein S14) to orchestrate ribosomal processing [23]. In order to elucidate if TcADKn behaves in a similar way as FAP7, we cloned Rps14 ortholog from *T. cruzi* (TcRps14, Systematic ID: Tc00.1047053506945.230) and realized protein interaction assays in vitro using the recombinant proteins, histidine-tagged TcADKn (6x-His-TcADKn) histidine-tagged arginine kinase (6x-His-TcAK), glutathione S-transferase tagged TcRps14 (GST-TcRps14) and the glutathione S-transferase epitope (GST). Results were analyzed by Western Blot, using monoclonal anti-GST and anti-HIS antibodies. As Figure 5B and C show, we could detect interaction between TcADKn and TcRps14 (Panel A, lane D) and not with the GST tag alone (Panel B, lane D) or with a non-related protein (TcAK) in vitro (Panel A, lane B). These results reinforce the hypothesis of TcADKn's participation in ribosome processing in a similar way as FAP7.

#### TcADKn is involved in ribosome biogenesis

In order to deepen in the possible role of TcADKn in ribosome biogenesis we decided to study 18S rRNA processing in *Trypanosoma cruzi*. During ribosome maturation, rRNA suffers several cleavages which are orchestrated by a large amount of proteins. If TcADKn was involved in ribosome biogenesis we expected to find ribosomal precursors in RNA extracted from immunoprecipitation assays against TcADKn. Due to the lack of data about ribosome processing in trypanosomatids we did not have information about ribosomal precursors nor processing sites. Using a strategy for isolating small RNAs we were able to clone the different 18S rRNA precursors from total RNA samples from *T. cruzi* epimastigotes (indicated as “+” in Figure 5D) and determine the end of the 18S subunit (indicated as “18” in Figure 5D). The same strategy was used to analyze the presence of ribosomal precursors in RNA extracted from TcADKn immunoprecipitation assays. We detected ribosomal precursors in the immunoprecipitate (indicated as “IP” in Figure 5D), which were absent in pre-immune extracts (indicated as “-” in Figure 5D), reinforcing TcADKn possible role in ribosome biogenesis (Figure 5D). Furthermore, we could not detect non-related mRNAs in immunoprecipitates nor amplification products in samples were no retrotranscriptase was added, eliminating any DNA contamination possibilities (Figure S5 A and B).

**Figure 6:**
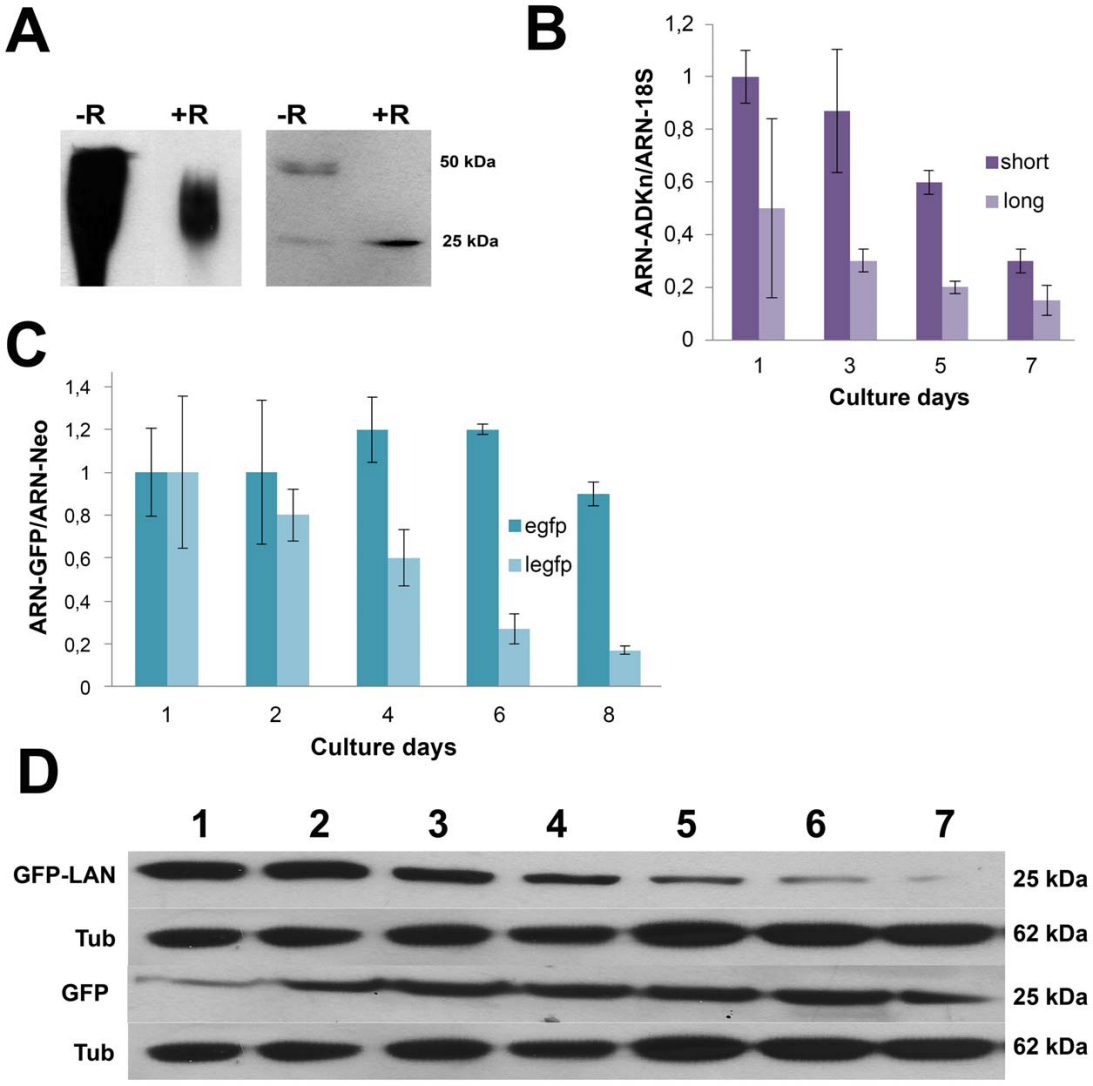
Regulation of TcADKn mRNA. A) TcADKn interaction with RNA 1×10^8^ epimastigotes in day 2 of culture grown in BHT medium were harvested, and resuspended in Tris-HCl buffer pH 7,4 and broken by 3 cycles of freezing and thawing. Native and denaturing 8% PAGE followed by Western Blot against TcADKn using samples of epimastigotes from day 2 of culture treated (+R) or not (−R) with RNAase I (10 mg.mL^−1^) for 2 h. at 37C. SDS- PAGE Band intensities were normalized using anti-Tubulin antibodies, quantified and represented using a bar graphic. B) Real time PCR quantifying the two mRNAs found for TcADKn along the epimastigotes growth curve. The value obtained for the short transcript in day one was taken as 1. Data were relativized to the 18S. C) Real time PCR along the epimastigotes growth curve of transgenic parasites expressing the pTREX-OMNI or pTREX-OMNI LAN (3′UTR) construction. The value obtained for GFP of the pTREX-OMNI construct was taken as 1. Data were relativized to the neo gene found in the expression vector. D) Western Blot analysis of GFP expression along the growth curve of parasites expressing pTREX-OMNI or pTREX-OMNI-LAN construction. Tubulin (TUB) was used as load control. Each lane corresponds to 4×10^6^ parasites.

Proteins involved in ribosome biogenesis interact with RNA precursors. To study if TcADKn interacts with RNA we incubated parasites extracts with RNAseI. Results were analyzed by Western Blot in native and denaturing gels (SDS-PAGE). As Figure 6A shows, the higher molecular weight band completely disappeared after RNAaseI treatment. In addition, TcADKn migrated differently under native conditions in treated samples. In conclusion, TcADKn could be interacting with RNA and that might be the cause of the higher apparent molecular weight band previously observed in Western Blotting. Furthermore, this interaction might be causing conformational changes similar to the ones that have been observed when these enzymes exert their ATPase or ADK activity. Another possibility is that the migration variation observed could be just a consequence of the interaction with RNA [71].

### Regulation of TcADKn expression levels

#### TcADKn expression is regulated by its 3′ UTR

In trypanosomatids gene expression is mainly regulated post transcriptionally at mRNA level. Several cases have been described in which sequences in the 3′ untranslated region (3′ UTR) of an mRNA play a key role in gene expression [72], [73]. Considering that TcADKn levels decrease along the epimastigote growth curve, we decided to study if its expression was regulated at an mRNA level. By reverse transcriptase PCR (RT-PCR) we were able to isolate two different mRNAs of 666 and 736 bases, which differed in their 3′ UTR. The abundance of both mRNA species along the epimastigotes growth curve was quantified by real time PCR. The shorter mRNA appeared in higher levels along the growth curve, at least 2-times fold with respect to the long mRNA, but both showed the same tendency, they decreased as parasites reached stationary phase (Figure 6B). These results indicate that TcADKn expression could be regulated at an mRNA level. As both mRNAs present different lengths they could be under the regulation of a different concert of proteins which maintain physiological levels of TcADKn.

The next issue we focused on was if TcADKn's 3′ UTR could regulate the expression of a reporter protein. So, we cloned the whole 3′ UTR down-stream of the eGFP coding gene in a single copy vector, we named this construction pTREX-OMNIeGFP-LAN, as a control vector we used the pTREX-OMNIeGFP vector. After obtaining stable transgenic parasite lines we quantified eGFP expression at protein and mRNA level. We found that eGFP levels decreased along the epimastigotes growth curve in parasites expressing the pTREX-eGFP-LAN construction, reaching 5-times fold lower levels than eGFP levels from parasites expressing the pTREX-OMNIeGFP construct (Figure 6 C, D). It is important to highlight that in this experiment we quantified total GFP levels, we did not discriminate the presence of different mRNAs as it happens in TcADKn. These observations indicate that TcADKn 3′ UTR could be the responsible of regulating its abundance along the parasites life cycle. What is more, this sequence is capable of regulating the expression of a non-related protein (the reporter gene eGFP) in a similar way.

## Discussion

Adenylate kinases have been mainly related to nucleotide interconversion and energy management. [74]. In 2005 the first nuclear ADK isoform was found [21], later they were characterized in several organisms [17], [18], [19]. This “atypical” isoform in terms of primary structure was associated to ribosomes biogenesis in yeast [23] and to Cajal bodies organization in humans [20], [27], [71]. In these enzymes the P-loop domain, responsible of nucleotide binding in phosphotransferases, could be involved in other functions such as protein interactions, endonucleolytic RNA cleavage, RNA-protein interaction or RNA metabolism [17], [18], [23], [28]. Even though the function of nuclear adenylate kinases is not completely understood it must be extremely important as they are essential for cell viability [17], [19], [23], [28].

In this paper we report the existence of the 7^th^ ADK variant in *T. cruzi*, which corresponds to the nuclear isoform. We studied its nuclear shuttling and characterized its non-canonical nuclear localization signal, being one of the few atypical NLS that involves the catalytic site of the protein (Walker domain or P- loop). Probably its enzymatic activity is not essential for its nuclear importation as fusion proteins did not yield a higher-than-expected-band in western blotting suggesting that they might be inactive. We postulate that TcADKn enters the nucleus in an unfolded conformation, being the nuclear localization signal within the P-loop, once it enters the nucleus it folds correctly regarding the active site inside the protein. The available data does not allow us to conclude how the importation process takes place; TcADKn could be forming a complex with other proteins, which are recognized by the importin and then enter the nucleus or it could be recognized directly by the importing complex. Further experiments should be carried out in order to understand the nuclear importation mechanism. We could also relate its nuclear exportation to the CRM1 exportin adapter [49], being one of the few proteins in *T. cruzi* which has been reported to use this transporter.

*T. cruzi* ribosomes have been studied for a long time because they exhibit unique characteristics which are absent in higher eukaryotes and that could be capitalized for therapeutic drug design [12]. Scientific studies have focused on ribosomal structure rather than in its biogenesis. In trypanosomatids there is almost no evidence about ribosome processing sites or the proteins involved in each step. Proteomic data has revealed that many ribosomal proteins and accessory non-ribosomal proteins are conserved in *T. cruzi* [11], however their function has not been determined [12]. TcADKn homolog in yeast has been related to ribosome processing, being associated to the final cytoplasmic step of maturation of the 18S rRNA by direct interaction with TcRps14. [23]. By yeast complementation assays we could postulate that TcADKn could be involved in ribosome 18S rRNA processing. This idea is reinforced with the fact that we detected in vitro interaction between TcADKn and TcRps14 and moreover we detected ribosomal precursors in TcADKn immunoprecipitates. These data suggest that ribosome biogenesis in *T. cruzi* presents conserved characteristics with yeast. However it also presents similar characteristics to mammals. In yeast the ribosomal precursors of the 18S rRNA subunit presents only one intermediary after A2 cleavage within the ITS1, while mammals present two intermediaries [75]. In our experiments we could detect two 18S pre-rRNA precursors indicating that the 18S rRNA processing presents both mammalian and yeast characteristics. So in *T. cruzi* 18S rRNA biogenesis would be unique as it combines characteristics of both mammals and yeast.

Finally, TcADKn nuclear shuttling is regulated by nutrient availability, ribosome biogenesis, DNA integrity, oxidative stress and probably by the equivalent of the mammalian TOR pathway in *T. cruzi*. Furthermore the results obtained after puromycin and cicloheximide treatment suggest that ribosome assembly might be necessary for TcADKn nucleolar localization as this one is lost when ribosomes cannot reassemble after cicloheximide treatment. On the contrary in puromycin treatment in which protein synthesis is blocked but ribosomes can re-assembly, nucleolar localization is not disrupted [56], [57], [58], [59]. Similar regulation mechanisms have been observed for other ribosomal proteins [76]. The existence of tight regulation mechanisms gives us an idea of the complexity of ribosome biogenesis and the susceptibility of this process to environmental changes and unfavorable conditions.

**Figure 7:**
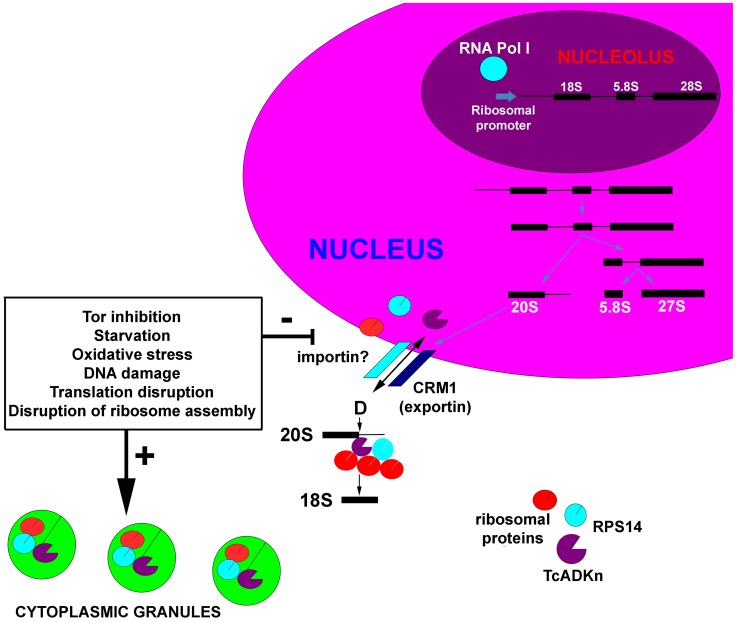
Model proposed for TcADKn function and regulation. TcADKn is involved in ribosome biogenesis, being involved in the processing of the 18S precursor at site D by directly interacting with TcRps14. TcADKn would be regulated by nutrient availability, ribosome activity, active transcription and the TOR pathway. The scheme represents a putative dynamic model for the role of TcADKn on ribosome biogenesis and its regulation.

Figure 7 summarizes the role of TcADKn in the formation of the 18S ribosomal subunit and its regulation mechanisms. We hope that the information presented encourages the study of ribosome biogenesis in these divergent organisms.

## Supporting Information

Figure S1 **Alignment of adenylate kinase sequences.** A) Global sequence alignment of adenylate kinases from *T. cruzi* (A) (TcADK1 Tc00.1047053506855.180, TcADK2 Tc00.1047053506195.90, TcADK3 Tc00.1047053509733.180, TcADK4 Tc00.1047053507057.20, TcADK5 Tc00.1047053510575.180, or TcADK6 Tc00.1047053506195.80) and TcADKn (Tc00.1047053507023.280).B) Global sequenca aligment of nuclear adenylate kinases from *T. cruzi* (Tc00.1047053507023.280).*T. brucei* (Tb927.6.3210), *L. major* (LmjF30.1890), *S. cereviciae* ( GI 851388), *H. sapiens* (GI 64061), *D. melanogaster* (GI 36379), *C. elegans* (GI 174511). Alignments were performed using the Clustal algorithm. The conserved lysine involved in the catalysis, the P-loop, the LID domain (region involved in ATP binding and of covering the phosphastes of the active site) and the putative nuclear importation and exportation signals are highlighted in the sequences.
(TIF)

Figure S2 **TcADKn antibodies specificity.** A) Similar subcellular localization patterns were observed using specific antibodies that recognize TcADKn and heterologous expression of GFP fusion proteins. A TcADKn localization in epimastigotes of *T. cruzi* grown along the growth curve. Parasites were grown BHT medium, starting from 10^6^ epimastigotes, samples were collected at day 1 of culture (CL1). TcADKn localization was followed by indirect immunofluorescence using specific anti-TcADKn antibodies generated in mice. Epimastigotes of *T. cruzi* were transfected with the construction pTexNe and fluorescence was followed by fluorescence microscopy. DNA was stained with DAPI. B) Western Blot analysis using anti-TcADKn antibodies and GFP antibodies, to study antibodies specificity in parasites expressing the pTexNe construction (GFP) and total epimastigote samples (WT). *T. cruzi* epimastigotes were grown in BHT medium, starting from 1×10^6^ total parasites; samples were collected daily for protein sample preparation. In each lane 4×10^6^ parasites were loaded. As it can be observed both antibodies recognize the fusion protein.
(TIF)

Figure S3 **TcADKn biochemical characterization.** A) For ADK activity, a sample of 50 µg of protein fraction was added to the reaction mixture (100 mM Tris-HCl buffer pH 7.5, 20 mM glucose, 5 mM MgCl_2_, 100 mM KCl, 2 mM dithiothreitol, 1 mM NADP+, 5 U.mL−1 hexokinase and 2 U.mL−1 glucose-6-phosphate dehydrogenase) in a cuvette to a volume of 0.5 mL. After 5 min at 35°C the reaction was started by the addition of a small volume of ADP to a final concentration of 10 mM, unless otherwise indicated. ADK activity was calculated by measuring the increase in absorbance at 340 nm that accompanied the reduction of NADP+ [30]. B) For ATPase activity a sample of 50 µg of protein was added to the reaction mixture (100 mM Tris-HCl, pH 7.5, 60 mM KCl, 5 mM MgCl_2_, 5 U.mL−1 of polynucleotide kinase, 5 U.mL−1 of lactate dehydrogenase, 20 mM phosphoenolpyruvate, 1 mM NADH) After 5 min at 35°C the reaction was started by the addition of a small volume of ATP. ATPase activity was calculated by measuring the decrease in absorbance at 430 nm that accompanied the oxidation of NADH. Measurements were converted to enzymatic activity using the NAD and NADH extinction coefficient.
(TIF)

Figure S4 **Viability control and selection of transformed yeast.** Gal HA-FAP7 strain was transformed with TcADKn, TcADKn(K20R), TbADKn, TbADKF (*T. brucei* mitochondrial isoform, F), *E. coli* ADK, TcADK6 (*T. cruzi* mitochondrial isoform, 6) and ScFAP7 and sowed in A. YNB galactose medium for selection and B. YPG medium for viability control.
(TIF)

Figure S5 **Immunoprecipitation controls.** A. DNaseI control, RT-PCR against ITS1 where lane 1 non MMLV- retrotranscriptase was added, lane 2 with MMLV-retrotranscriptase. No amplification was observed in lane A discarding possible DNA contaminations. B Non-related mRNAs control. RT-PCR against TcNDPK3 (Systematic ID: Tc00.1047053510879.210) and TcH2B (Systematic ID: Tc00.1047053511635.20) in total parasites (lanes 1 and 3) and cDNA from TcADKn immunoprecipitates (lanes 2 and 4). No amplification bands were observed in the immunoprecipitates.
(TIF)

Table S1 **Analysis of adenylate kinases.** Similarities and identities of nuclear adenylate kinases from different organisms and different ADK isoforms from *T. cruzi* were calculated using the sequence alignment tool from the Vector NTI program (Invitrogen). (TcADK1: Tc00.1047053506855.180, TcADK2: Tc00.1047053506195.90, TcADK3: Tc00.1047053509733.180, TcADK4: Tc00.1047053507057.20, TcADK5: Tc00.1047053510575.180, or TcADK6: Tc00.1047053506195.80) or *T. cruzi* (Tc00.1047053507023.280), *T. brucei* (Tb927.6.3210), *L. major* (LmjF30.1890), *S. cereviciae* (GI 851388), *H. sapiens* (GI 64061), *D. melanogaster* (GI 36379), *C. elegans* (GI 174511).
(DOC)

Table S2 **Primers.** All primers mentioned in the text used for NLS mapping, Real time PCR, RNA isolation and for general purposes are listed.
(DOC)
