# Supplementary figures and images for: Molecular and Functional Characterization of a Trypanosoma cruzi Nuclear Adenylate Kinase Isoform
Source: PLoS Negl Trop Dis. 2013 Feb 7;7(2):e2044. doi: 10.1371/journal.pntd.0002044 (PMC3567042; doi:10.1371/journal.pntd.0002044)

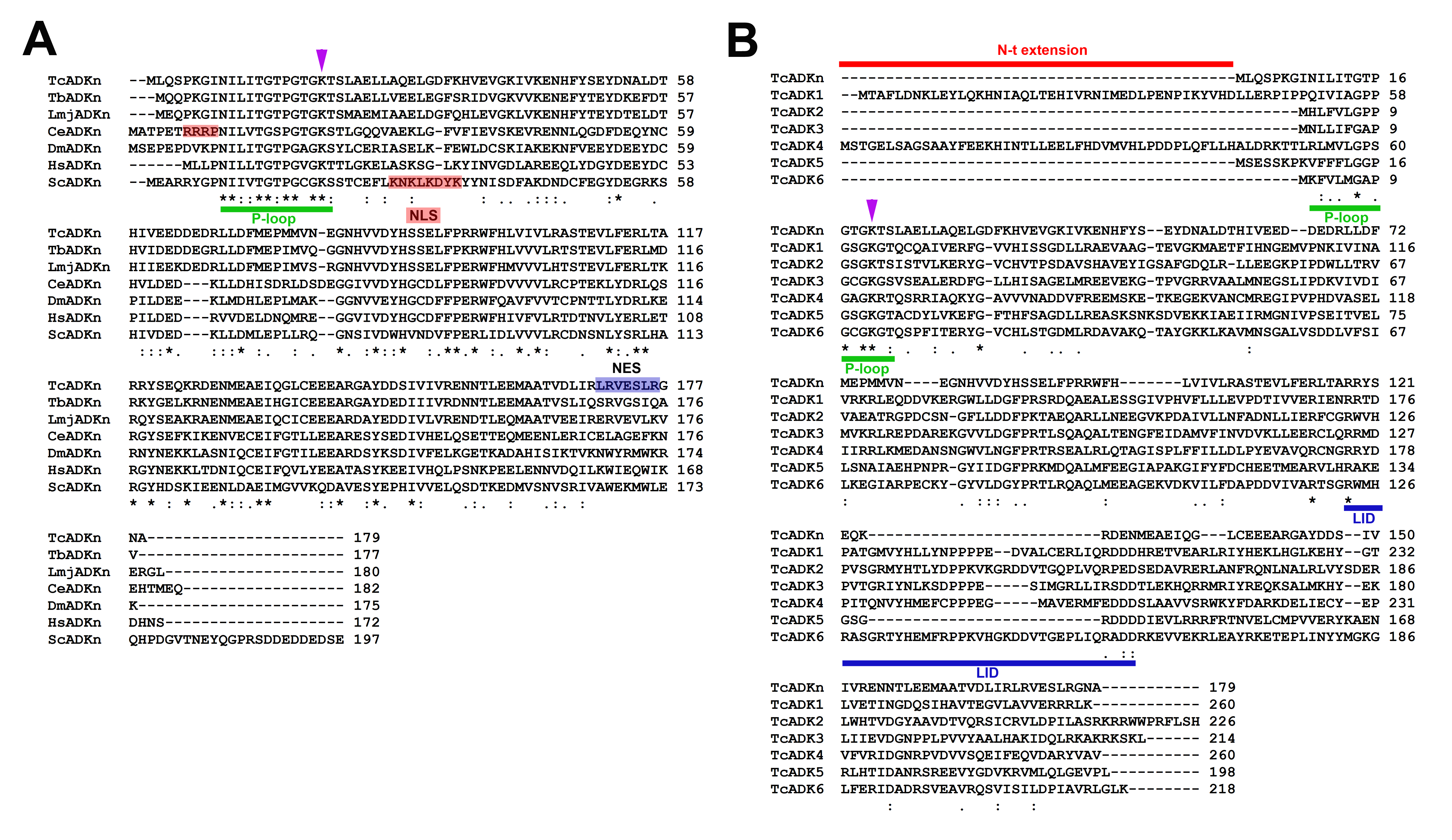

Supplement: Figure S1 — Alignment of adenylate kinase sequences. A) Global sequence alignment of adenylate kinases from T. cruzi (A) (TcADK1 Tc00.1047053506855.180, TcADK2 Tc00.1047053506195.90, TcADK3 Tc00.1047053509733.180, TcADK4 Tc00.1047053507057.20, TcADK5 Tc00.1047053510575.180, or TcADK6 Tc00.1047053506195.80) and TcADKn (Tc00.1047053507023.280).B) Global sequenca aligment of nuclear adenylate kinases from T. cruzi (Tc00.1047053507023.280).T. brucei (Tb927.6.3210), L. major (LmjF30.1890), S. cereviciae ( GI 851388), H. sapiens (GI 64061), D. melanogaster (GI 36379), C. elegans (GI 174511). Alignments were performed using the Clustal algorithm. The conserved lysine involved in the catalysis, the P-loop, the LID domain (region involved in ATP binding and of covering the phosphastes of the active site) and the putative nuclear importation and exportation signals are highlighted in the sequences. (TIF) [file pntd.0002044.s001.tif]

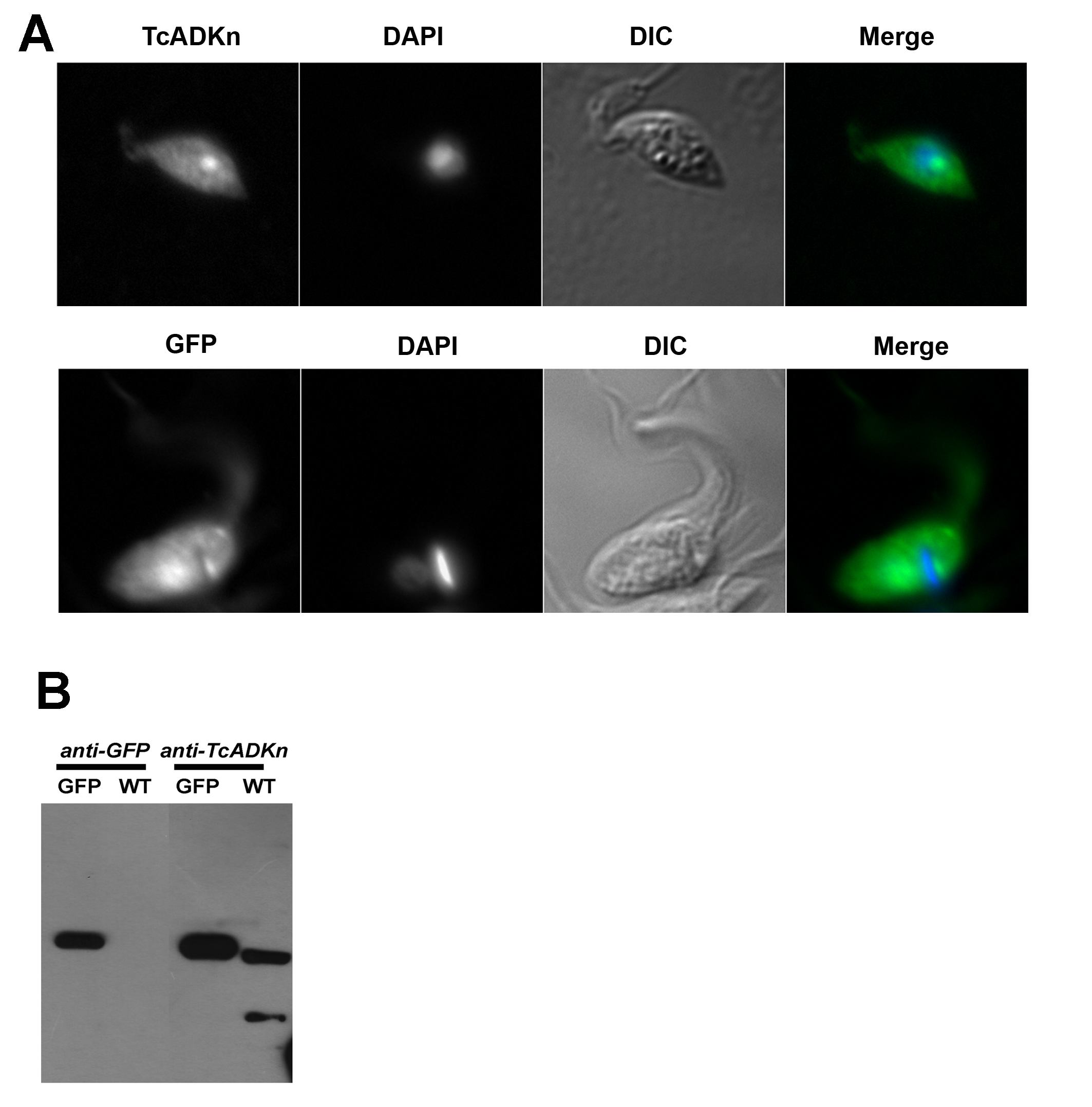

Supplement: Figure S2 — TcADKn antibodies specificity. A) Similar subcellular localization patterns were observed using specific antibodies that recognize TcADKn and heterologous expression of GFP fusion proteins. A TcADKn localization in epimastigotes of T. cruzi grown along the growth curve. Parasites were grown BHT medium, starting from 106 epimastigotes, samples were collected at day 1 of culture (CL1). TcADKn localization was followed by indirect immunofluorescence using specific anti-TcADKn antibodies generated in mice. Epimastigotes of T. cruzi were transfected with the construction pTexNe and fluorescence was followed by fluorescence microscopy. DNA was stained with DAPI. B) Western Blot analysis using anti-TcADKn antibodies and GFP antibodies, to study antibodies specificity in parasites expressing the pTexNe construction (GFP) and total epimastigote samples (WT). T. cruzi epimastigotes were grown in BHT medium, starting from 1×10^6^ total parasites; samples were collected daily for protein sample preparation. In each lane 4×10^6^ parasites were loaded. As it can be observed both antibodies recognize the fusion protein. (TIF) [file pntd.0002044.s002.tif]

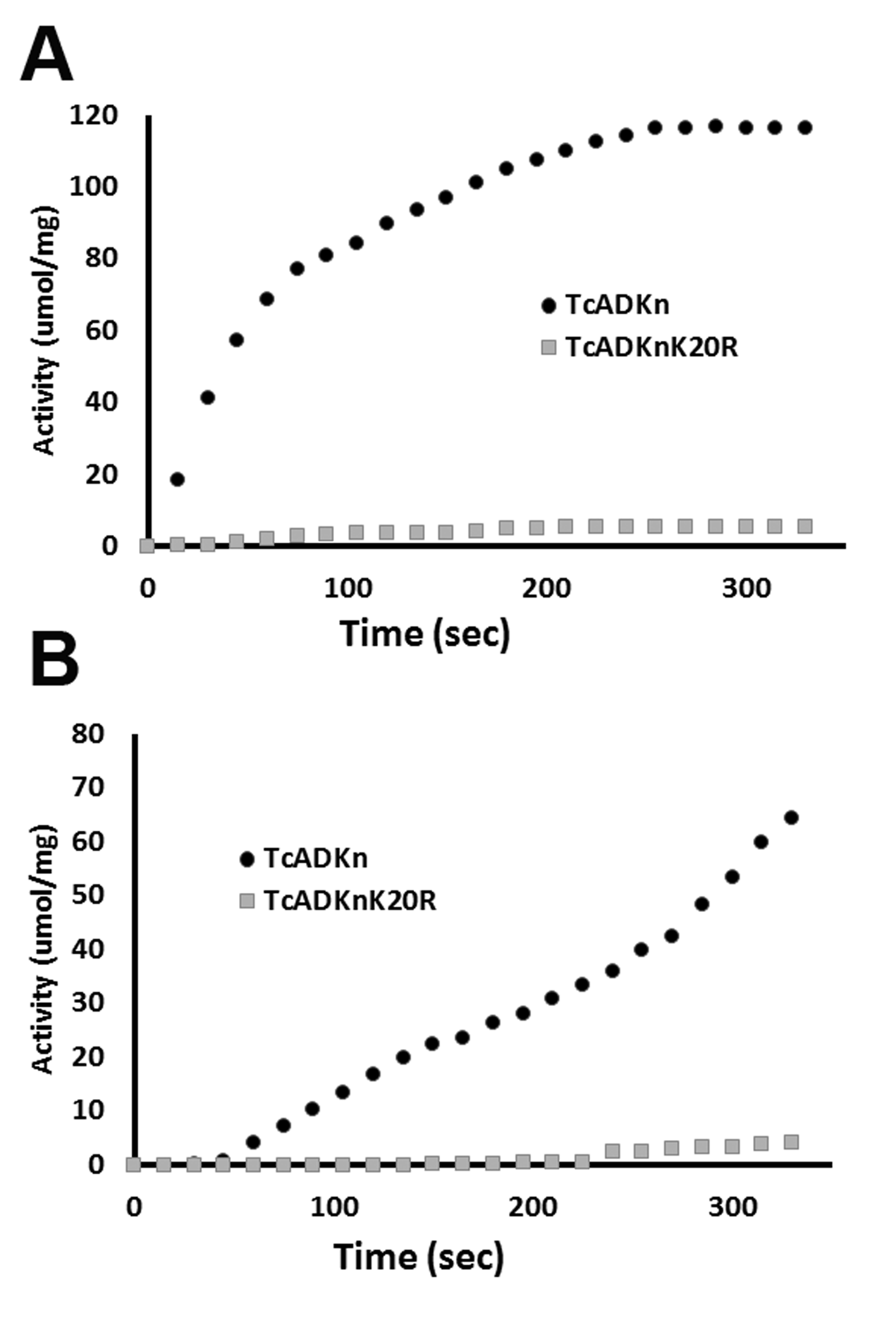

Supplement: Figure S3 — TcADKn biochemical characterization. A) For ADK activity, a sample of 50 µg of protein fraction was added to the reaction mixture (100 mM Tris-HCl buffer pH 7.5, 20 mM glucose, 5 mM MgCl2, 100 mM KCl, 2 mM dithiothreitol, 1 mM NADP+, 5 U.mL−1 hexokinase and 2 U.mL−1 glucose-6-phosphate dehydrogenase) in a cuvette to a volume of 0.5 mL. After 5 min at 35°C the reaction was started by the addition of a small volume of ADP to a final concentration of 10 mM, unless otherwise indicated. ADK activity was calculated by measuring the increase in absorbance at 340 nm that accompanied the reduction of NADP+ [30]. B) For ATPase activity a sample of 50 µg of protein was added to the reaction mixture (100 mM Tris-HCl, pH 7.5, 60 mM KCl, 5 mM MgCl2, 5 U.mL−1 of polynucleotide kinase, 5 U.mL−1 of lactate dehydrogenase, 20 mM phosphoenolpyruvate, 1 mM NADH) After 5 min at 35°C the reaction was started by the addition of a small volume of ATP. ATPase activity was calculated by measuring the decrease in absorbance at 430 nm that accompanied the oxidation of NADH. Measurements were converted to enzymatic activity using the NAD and NADH extinction coefficient. (TIF) [file pntd.0002044.s003.tif]

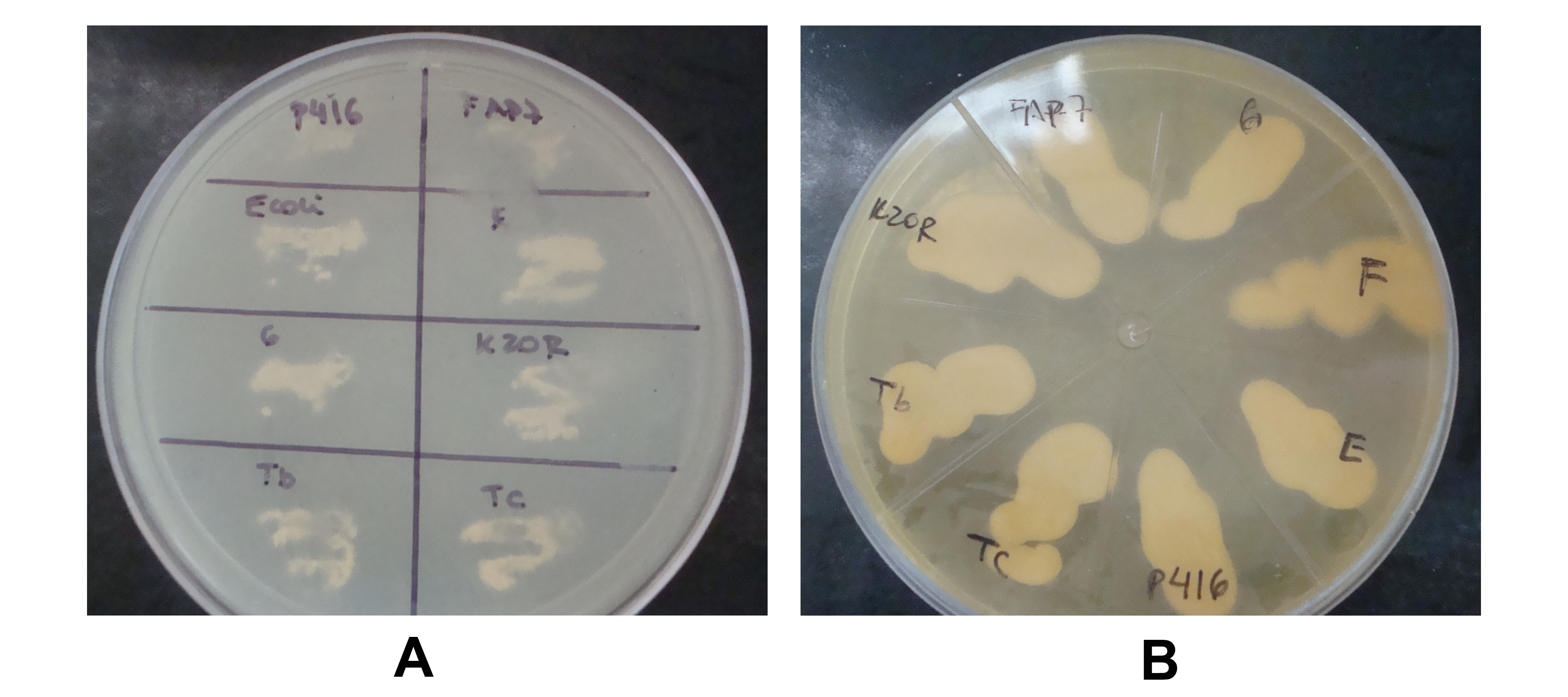

Supplement: Figure S4 — Viability control and selection of transformed yeast. Gal HA-FAP7 strain was transformed with TcADKn, TcADKn(K20R), TbADKn, TbADKF (T. brucei mitochondrial isoform, F), E. coli ADK, TcADK6 (T. cruzi mitochondrial isoform, 6) and ScFAP7 and sowed in A. YNB galactose medium for selection and B. YPG medium for viability control. (TIF) [file pntd.0002044.s004.tif]

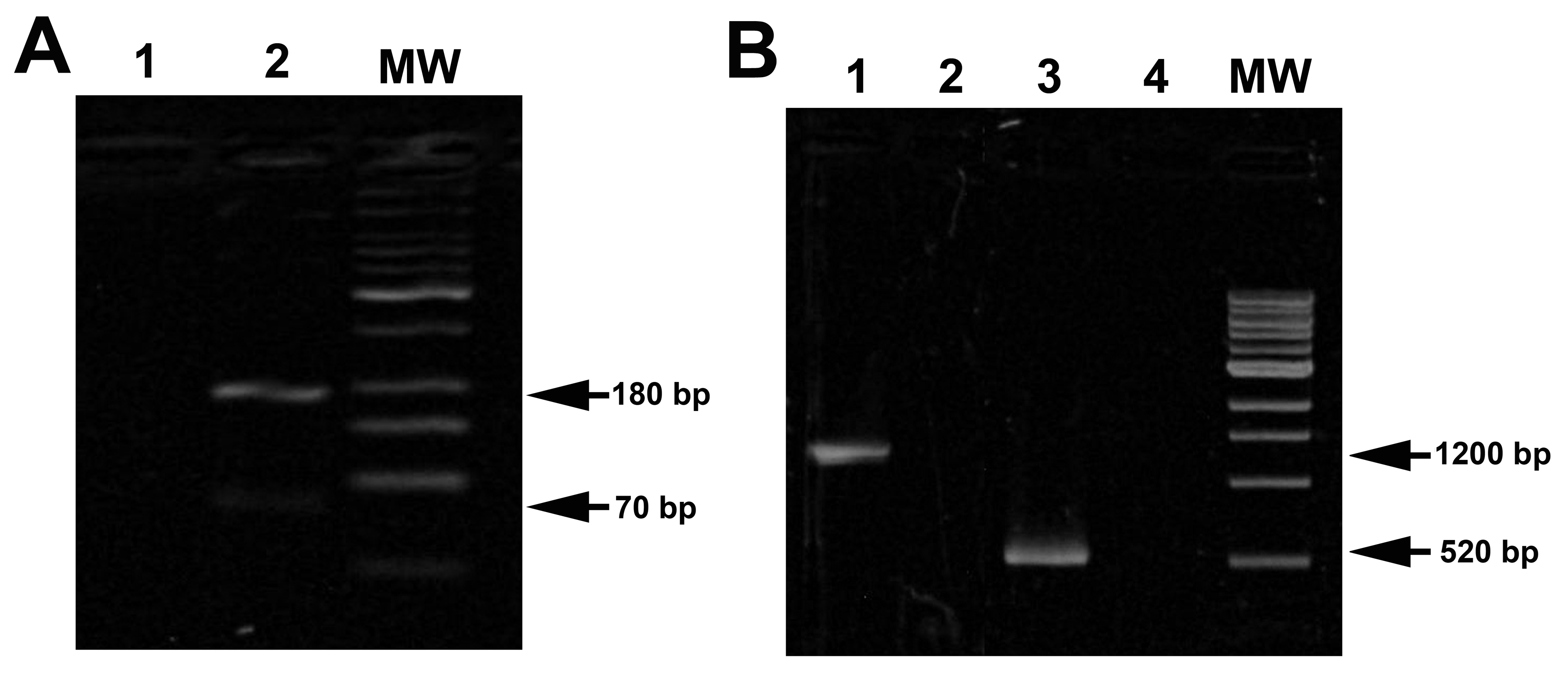

Supplement: Figure S5 — Immunoprecipitation controls. A. DNaseI control, RT-PCR against ITS1 where lane 1 non MMLV- retrotranscriptase was added, lane 2 with MMLV-retrotranscriptase. No amplification was observed in lane A discarding possible DNA contaminations. B Non-related mRNAs control. RT-PCR against TcNDPK3 (Systematic ID: Tc00.1047053510879.210) and TcH2B (Systematic ID: Tc00.1047053511635.20) in total parasites (lanes 1 and 3) and cDNA from TcADKn immunoprecipitates (lanes 2 and 4). No amplification bands were observed in the immunoprecipitates. (TIF) [file pntd.0002044.s005.tif]
