# Supplementary material for: Molecular and Functional Characterization of a Trypanosoma cruzi Nuclear Adenylate Kinase Isoform
Source: PLoS Negl Trop Dis. 2013 Feb 7;7(2):e2044. doi: 10.1371/journal.pntd.0002044 (PMC3567042; doi:10.1371/journal.pntd.0002044)
Supplement: Table S1 — Analysis of adenylate kinases. Similarities and identities of nuclear adenylate kinases from different organisms and different ADK isoforms from T. cruzi were calculated using the sequence alignment tool from the Vector NTI program (Invitrogen). (TcADK1: Tc00.1047053506855.180, TcADK2: Tc00.1047053506195.90, TcADK3: Tc00.1047053509733.180, TcADK4: Tc00.1047053507057.20, TcADK5: Tc00.1047053510575.180, or TcADK6: Tc00.1047053506195.80) or T. cruzi (Tc00.1047053507023.280), T. brucei (Tb927.6.3210), L. major (LmjF30.1890), S. cereviciae (GI 851388), H. sapiens (GI 64061), D. melanogaster (GI 36379), C. elegans (GI 174511). (DOC) [file pntd.0002044.s006.doc]

|  | Dm | | Tb | | Tc | | Sc | | Hs | | Lm | | Ce | |
| --- | --- | --- | --- | --- | --- | --- | --- | --- | --- | --- | --- | --- | --- | --- |
|  | Similarity | Similarity | Similarity | Identity | Similarity | Identity | Similarity | Identity | Similarity | Identity | Similarity | Identity | Similarity | Identity |
| Dm |  |  | 51,1 | 37,4 | 49,2 | 36,1 | 46,5 | 34 | 57,9 | 43,8 | 53,3 | 39,3 | 59,3 | 45,6 |
| Tb |  |  |  |  | 83,3 | 73,2 | 47,2 | 31,1 | 53,3 | 39,4 | 79,4 | 70,1 | 51,9 | 40 |
| Tc |  |  |  |  |  |  | 49,5 | 37,2 | 54,1 | 40,3 | 82,9 | 73,5 | 54,8 | 38,7 |
| Sc |  |  |  |  |  |  |  |  | 48,5 | 34,3 | 46,5 | 30,7 | 49,5 | 36,6 |
| Hs |  |  |  |  |  |  |  |  |  |  | 53,9 | 38,7 | 59,9 | 45,1 |
| Lm |  |  |  |  |  |  |  |  |  |  |  |  | 57,7 | 41,9 |
| Ce |  |  |  |  |  |  |  |  |  |  |  |  |  |  |

Dm *Drosophila melanogaster*

Tb *Trypanosoma brucei*

Tc *Trypanosoma cruzi*

Sc *Saccharomyces cerevisiae*

Hs *Homo sapiens*

Lm *Leishmania major*

Ce *Caenorhabditis elegans*

|  | adk1 | | adk2 | | adk3 | | adk4 | | adk5 | | adk6 | | TcADKn | |
| --- | --- | --- | --- | --- | --- | --- | --- | --- | --- | --- | --- | --- | --- | --- |
|  | Similarity | Identity | Similarity | Identity | Similarity | Identity | Similarity | Identity | Similarity | Identity | Similarity | Identity | Similarity | Identity |
| adk1 |  |  | 34,2 | 21,9 | 43,3 | 28,8 | 44,8 | 27,7 | 30,2 | 21,3 | 40,5 | 27,8 | 24,1 | 13,4 |
| adk2 |  |  |  |  | 43,3 | 27,6 | 30,4 | 19 | 29 | 15,9 | 50,2 | 32,5 | 25,3 | 11,8 |
| adk3 |  |  |  |  |  |  | 38,2 | 24 | 38,9 | 22,1 | 48,8 | 28 | 31,3 | 16,1 |
| adk4 |  |  |  |  |  |  |  |  | 24,8 | 15,6 | 34,1 | 22,3 | 19,9 | 11,9 |
| adk5 |  |  |  |  |  |  |  |  |  |  | 39,2 | 23,7 | 30,2 | 16,8 |
| adk6 |  |  |  |  |  |  |  |  |  |  |  |  | 22,8 | 12,5 |
| TcADKn |  |  |  |  |  |  |  |  |  |  |  |  |  |  |
