# Supplementary material for: Molecular and Functional Characterization of a Trypanosoma cruzi Nuclear Adenylate Kinase Isoform
Source: PLoS Negl Trop Dis. 2013 Feb 7;7(2):e2044. doi: 10.1371/journal.pntd.0002044 (PMC3567042; doi:10.1371/journal.pntd.0002044)
Supplement: Table S2 — Primers. All primers mentioned in the text used for NLS mapping, Real time PCR, RNA isolation and for general purposes are listed. (DOC) [file pntd.0002044.s007.doc]

GENERAL PRIMERS:

| construction | primer F | sequence | site | primer R | sequence | site | vector |
| --- | --- | --- | --- | --- | --- | --- | --- |
| Rps14 | Rps14F | AAGCTTATGTCGAAGAAGCAGGAAGTG | HindIII | Rps14R | GGATCCCTACAGACGACGACCACGGC | BamHI | pGEX |
| pRSETADKn | ADKNF | AAGCTTGATGTTGCAGAGTCCG | HindIII | ADKNR | AATCGGGTGATAATCCACAACGT | XhoI | PRSET |
| pRSETADKnK20R | ADKNF | AAGCTTGATGTTGCAGAGTCCG |  | ADKNR | AATCGGGTGATAATCCACAACGT | - | PRSET |
| PTREXOMNILAN | LANF | CAATT GCATGAAGTTGCGTGACATGG | MfeI | LANR | CTCG AGGCCCGGTAATCTTCCTCTG | XhoI | PTREXOMNI |
| p416TcADKn | ADKNF | AAGCTTGATGTTGCAGAGTCCG | HindIII | ADKNR | AATCGGGTGATAATCCACAACGT | XhoI | p416 |
| P416TcADKnK20R | ADKNF | AAGCTTGATGTTGCAGAGTCCG | HindIII | ADKNR | AATCGGGTGATAATCCACAACGT | XhoI | p416 |
| P416TbADKn | tbADKNF | CTAGAATGCAACAGCCG | XbaI | tbADKNR | AATCGGGCCATGAATTTCAGCC | XhoI | p416 |
| P416FAP7 | FAP7F | GAATTC ATGGAAGCAAGACGGTATGG | EcoRI | FAP7R | CTCGAG CTACTCACTGTCTTCATCGT | XhoI | p416 |
| P416Ecoli | ECF | TTTGGATCCATGCGTATCATTCTGCTTGG | BamHI | ECR | TTCAAGCTTAGCCGAGAATTTTTTCCA | HindIII | p416 |
| P416ADK6 | ADK6F | GGATCCATGAAGTTTGTTTTGATGGGAG | BamHI | ADK6R | AAGCTTCCAGAGCACTATTTGAGCCC | HindIII | p416 |
| P416ADKF | ADKFF | CCGAAGCTTATGAAGGTAATTTTTCTTGGACCTCC | BamHI | ADKFR | TAAGGTACCTTAATTCCCATGTATCTTTGCACCG | HindIII | p416 |
| p416hCINAP | hCINAPF | GGATCC ATGTTGCTTCCGAACATCCTGCTCAC | BamHI | hCINAPR | AAGCTT TGGGCAGCTGATGCACGATT | HindIII | p416 |

NLS MAPPING:

| Ne | ADKNF | HindIII | AAGCTTGATGTTGCAGAGTCCG | ADKNR | GTCGACCGACGCTGCATTACCCCTCA | SalI | PTEX |
| --- | --- | --- | --- | --- | --- | --- | --- |
| Nt | ADKNF | HindIII | AAGCTTGATGTTGCAGAGTCCG | ADKNNTR | GGATCCAATTTTCCCCACCT | BamHI | PTEX |
| eN | ADKNF | HindIII | AAGCTTGATGTTGCAGAGTCCG | ADKNR | GTCGACCGACGCTGCATTACCCCTCA | SalI | PTEX |
| Ct | ADKNCTF | EcoRI | GAATTC GTGAAGGAGAATCACTTCTACAGCGAGTA | EGFR | AAGCTTTTGTACAGCTCGTCCATGCC | HindIII | PTEX |
| NtNt | ADKNF | HindIII | AAGCTTGATGTTGCAGAGTCCG | ADKNNTR | GGATCCAATTTTCCCCACCT | BamHI | PTEX |
| CtNt | ADKNctF | EcoRI | GAATTC GTGAAGGAGAATCACTTCTACAGCGAGTA | EGFR | AAGCTTTTGTACAGCTCGTCCATGCC |  | PTEX |
| deltaploop | sploopF | EcoRI | GAATTCATGACATCTCTCGCTGAGCTTCTTAC | EGFR | AAGCTTTTGTACAGCTCGTCCATGCC | HindIII | PTEX |
| K20R | ADKNF | HindIII | AAGCTTGATGTTGCAGAGTCCG | ADKNR | GTCGACCGACGCTGCATTACCCCTCA | SalI | PTEX |
|  | ANK2RF |  | GGA ACA GGC AGG ACC TCT CTT GCT G | ANK2RR | CAG CAA GAG AGG TCC TGC CTG TTC C |  | PTEX |
| deltaNES | ADKNF | HindIII | AAGCTTGATGTTGCAGAGTCCG | ADKNDNES | GGATCCTTCAAGAGTGTTGTTTTCCCG | BamHI | PTEX |
| deltaNESK20R | ADKNF | HindIII | AAGCTTGATGTTGCAGAGTCCG | ADKNDNES | GGATCCTTCAAGAGTGTTGTTTTCCCG | BamHI | PTEX |

REAL TIME:

| primer | | sequence | | | | | primer | sequence | | | | |  | |
| --- | --- | --- | --- | --- | --- | --- | --- | --- | --- | --- | --- | --- | --- | --- |
| UTR1F | | GTTCTCTTTGAGCGTCTCAC | | | | | UTR1R | CGCAGACGTATCAAATCCAC | | | | |  | |
| uTR2F | | GACTGTGGATTTGATACGTCTG | | | | | UTR2R | TTAATGTTGCCTTTGGAGGT | | | | |  | |
| 18SF | | ATACCTTCCTCAATCAAGAACC | | | | | 18SR | AAATAATCAAACCCGACCAC | | | | |  | |
| GFPF | | ACCATCTTCTTCAAGGACGA | | | | | GFPR | GGCTGTTGTAGTTGTACTCC | | | | |  | |
| NEOF | | CTATCAGGACATAGCGTTGG | | | | | NEOR | CAGAAGAACTCGTCAAGAAGG | | | | |  | |
|  |  | |  |  | | | | | | |  |  | | |
|  |  | |  |  | | | | | | |  |  | | |
|  |  | |  |  | | | | | | |  |  | | |
|  | mRNA isolation | | |  | | | | | | |  |  | | |
|  |  | |  |  | | | | | | |  |  | | |
|  | primer | | sequence | | primer | | | | sequence | | | | |  |
| 5'UTR | SL | | AACGCTATTATTGATACAGTTTCTGTACTATATTG | | ADKNNTR | | | | GGATCCAATTTTCCCCACCT | | | | |  |
| 3'UTR | L3ANFX | | CTCGAGGGAAAACAACACTCTTGAAGAAA | | oligodT | | | | GGGACAGGGTTTTTTTTTTTTTTTV | | | | |  |
| LocusIP | L3ANFX | | CTCGAGGGAAAACAACACTCTTGAAGAAA | | LR | | | | GATTGATGGTGCCTACAG | | | | |  |
|  |  | |  |  | | | | | | |  |  | | |
|  |  | |  |  | | | | | | |  |  | | |
|  |  | |  |  | | | | | | |  |  | | |
|  | cloning of 18S precursors | | |  | | | | | | |  |  | | |
|  |  | |  |  | | | | | | |  |  | | |
|  | primer | | sequence | | | primer | | | |  | | | |  |
| adapter | LF | | Phosphorylation-CTGTAGGCACCATCAATC-ddC | | |  | | | |  | | | |  |
| cDNA | LR | | GATTGATGGTGCCTACAG | | |  | | | |  | | | |  |
| 18S | 18S ITSF | | GGCGGTATTCGCTTGTATCCT | | | LR | | | | GATTGATGGTGCCTACAG | | | |  |
| ETS | ETSF | | GGAATCACGTAGACCCAC | | | LR | | | | GATTGATGGTGCCTACAG | | | |  |
| NdPK3 | N3F | | GCTACATGGCGTATCGAGGCAGG | | | LR | | | | GATTGATGGTGCCTACAG | | | |  |
| H2B | H2BF | | GATCCACTAGGGCGTGCGACACCG | | | LR | | | | GATTGATGGTGCCTACAG | | | |  |
